# Identifying Policy-relevant Indicators for Assessing Landscape Vegetation Patterns to Inform Planning and Management on Multiple-use Public Lands

**DOI:** 10.1007/s00267-021-01493-8

**Published:** 2021-06-26

**Authors:** Sarah K. Carter, L. E. Burris, Christopher T. Domschke, Steven L. Garman, Travis Haby, Benjamin R. Harms, E. Kachergis, S. E. Litschert, Kevin H. Miller

**Affiliations:** 1 Bureau of Land Management, Colorado State Office, 2850 Youngfield St, Lakewood, CO 80215 USA; 2 Bureau of Land Management, National Operations Center, Denver Federal Center, Bldg. 50, P.O. Box 25047, Denver, CO 80225-0047 USA; 3 Quantum Spatial Inc., on contract to Bureau of Land Management, National Operations Center, Denver Federal Center, Bldg. 50, P.O. Box 25047, Denver, CO 80225-0047 USA; 4 U.S. Forest Service, 240W Prospect Rd., Fort Collins, CO 80526 USA; 5 Present Address: U.S. Geological Survey, Fort Collins Science Center, 2150 Centre Ave. Building C, Fort Collins, CO 80526-8118 USA; 6 Present Address: US Geological Survey, Denver Federal Center, Bldg. 25, Denver, CO 80225 USA

**Keywords:** Bureau of land management, LANDFIRE, Land health, Landscape approach, Landscape conservation, Landscape metrics

## Abstract

Understanding the structure and composition of landscapes can empower agencies to effectively manage public lands for multiple uses while sustaining land health. Many landscape metrics exist, but they are not often used in public land decision-making. Our objectives were to (1) develop and (2) apply a process for identifying a core set of indicators that public land managers can use to understand landscape-level resource patterns on and around public lands. We first developed a process for identifying indicators that are grounded in policy, feasible to quantify using existing data and resources, and useful for managers. We surveyed landscape monitoring efforts by other agencies, gathered science and agency input on monitoring goals, and quantified the prevalence of potential indicators in agency land health standards to identify five landscape indicators: amount, distribution, patch size, structural connectivity, and diversity of vegetation types. We then conducted pilot applications in four bureau of land management (BLM) field offices in Arizona, California, and Colorado to refine procedures for quantifying the indicators and assess the utility of the indicators for managers. Results highlighted the dominance of upland and the limited extent of riparian/wetland vegetation communities, moderate connectivity of priority vegetation patches, and lower diversity of native vegetation types on BLM compared to non-BLM lands. Agency staff can use the indicators to inform the development of quantitative resource management objectives in land use plans, evaluate progress in meeting those objectives, quantify potential impacts of proposed actions, and as a foundation for an all-lands approach to landscape-level management across public lands.

## Highlights

- Five landscape indicators are relevant to existing policy for multiple-use public lands.
- We quantified the indicators for priority vegetation types in three western states.
- Landscape indicators can inform objectives in land use plans for public lands.
- Indicator products must fit agency workflows and reporting requirements to be used.
- Limitations of current landcover data impede landscape monitoring on public lands.

## Introduction

There is a growing understanding that successful, long-term resource management and conservation often require prioritizing, coordinating, and implementing actions at landscape scales (e.g., Margules and Sarkar 2007; Lindenmayer et al. 2008; Sayer 2009; Clement et al. 2014; Carter et al. 2020). Landscape approaches to resource management can help achieve sustainable, multifunctional landscapes by considering landscape scales and using resource information collected at multiple spatial scales that is understandable and accessible to all stakeholders (Sayer et al. 2013; Freeman et al. 2015; Carter et al. 2016).

A landscape approach to resource management is particularly relevant for public lands. In the western United States (US), public lands are extensive and have complex and intertwined land ownership patterns (e.g., local, state, and federally managed lands are interspersed with private lands). Many public lands are managed for multiple uses (e.g., Federal Land Policy and Management Act of 1976 [43 USC §1701]) and are under pressure to provide diverse products and values to society, including oil and natural gas and diverse recreational opportunities (US Department of the Interior 2017a, 2018a). Management agencies are also charged with sustaining the health, diversity, productivity, and ecological integrity of public lands (Federal Land Policy and Management Act of 1976, Multiple-Use Sustained-Yield Act of 1960 [16 USC §528], Fundamentals of Rangeland Health [43 CFR §4180.1]). Goals to accommodate activities such as energy development, grazing, and recreation can conflict with goals to conserve species and maintain land health. Thus, it is critical for public land managers to understand the potential effects of different planning and management decisions on resource conditions within and across landscapes.

Management actions affect resource conditions at different spatial scales. Some disturbances affect resources locally (e.g., the footprint of a single natural gas well), while others affect resource patterns and conditions across large areas and over longer time periods (e.g., permitting right-of-way corridors, development of large natural gas fields). Disturbances large and small, and their associated direct and indirect effects on people, wildlife, and resources at multiple spatial and temporal scales, all contribute to cumulative effects of past, present, and reasonably foreseeable future actions, land uses, and associated human impacts that can significantly alter landscapes (e.g., Leu et al. 2008; Venter et al. 2016). As a result, managing public lands effectively for both multiple uses and sustained yield of natural resources requires assessing and monitoring resource patterns and conditions at multiple, including broad, spatial scales.

Monitoring is currently the least developed aspect of landscape approaches to resource management (Reed et al. 2016). Assessment and monitoring of natural resources on public lands have traditionally occurred on a project-by-project (site-by-site) basis in association with individual permits or projects (Wood et al. 2016), or more recently, across larger management units using survey sampling across field plots (Fancy and Bennetts 2012; Toevs et al. 2011b). Such design-based sampling efforts provide critical information on resource conditions locally, and sample measures can be aggregated to provide condition estimates across broader extents (e.g., Karl et al. 2016; Yu et al. 2020). Monitoring of vegetation species presence and density on small field sites, however, is not intended to provide information on the broad spatial patterns of vegetation across landscapes. Thus there is a need to identify a core set of landscape indicators that are relevant to public lands policy, are easy for land managers and the public to understand and interpret, and capture key information on resource patterns that managers can use to inform and evaluate the effectiveness of their planning and management actions.

A large body of research exists on the importance of understanding and monitoring landscape-scale resource patterns as part of a comprehensive assessment of resource condition (e.g., Li and Wu 2004; Riitters et al. 1995; Schindler et al. 2008; Uuemaa et al. 2009; Wickham et al. 1999) and for sustaining species, healthy landscapes, and protected area values (e.g., Environmental Protection Agency 2002, 2004; Mairota et al. 2013; Olsen et al. 2007; Soverel et al. 2010). Many landscape metrics have been developed to evaluate these patterns (e.g., Gustafson 1998; McGarigal et al. 2012). Researchers have explored which groups of metrics may characterize patterns most efficiently (e.g., Cushman et al. 2008) across different landscapes (e.g., Li et al. 2005; Szabo et al. 2014; Vaz et al. 2014), ecological levels (e.g., Wang and Yang 2012), spatial scales (e.g., Lustig et al. 2015; Wu et al. 2002), spatial extents (e.g., Hassett et al. 2012), and landcover classification schemes (e.g., Huang et al. 2006; Mas et al. 2010). The challenge is in bridging the gap between this research on landscape metrics and the use of landscape metrics by land managers.

There is a strong governmental commitment to science-informed, landscape-level management of public lands under federal jurisdiction in the US (BLM 2008a, 2009, 2012; Clement et al. 2014; Kitchell et al. 2015; US Department of the Interior 2021), and an acknowledgment that resource monitoring at landscape scales is needed to fully understand management impacts on resource condition (Taylor et al. 2014). Land use plans and policies lay the foundation for subsequent management decisions, including identifying which types of activities may be authorized in which areas (BLM 2005). Some land-use plans for public lands consider landscape ecology concepts such as fragmentation and connectivity (Trammell et al. 2018). Resource managers also see clear value in understanding the landscape context of decisions and assessments conducted at local levels (e.g., Wood et al. 2016). However, regular use of landscape metrics to inform planning and management decisions on public lands is lacking.

Our goal was to work to bridge the research-management gap specifically as it relates to landscape indicators and their use in decision-making on multiple-use public lands. Our objectives were to (1) develop and (2) apply a process for identifying a core set of landscape indicators that public land managers could use to assess, and ultimately monitor, resource patterns within and across landscapes in the western US. We define core indicators as indicators that quantify measurable ecosystem components that are applicable across ecosystems, management objectives, and agencies (BLM 2015a). We define landscape as an area encompassing an interacting mosaic of ecosystems and human systems characterized by a set of common management concerns (Clement et al. 2014). We use the term landscape indicator to refer to aspects of landscape pattern that we want to measure, and the term landscape metric to refer to the specific (calculated) values for the indicators using the stated methods. We ultimately quantify the landscape indicators only for vegetation types in this work, but continue to use the term landscape indicators throughout for simplicity and because the indicators may be relevant to quantifying patterns of other resources (e.g., soil types). Although our intent was to provide a generalized schema for public lands, we worked closely with the bureau of land management (BLM) in developing our process to help ensure it was practical, useful, and relevant for the agency, which administers circa one-eighth of the US landmass (BLM 2019a). We also conducted pilot applications in four BLM field offices to help refine the procedures to derive and quantify the indicators, and to better understand the utility of the indicators for managers.

Below we first present information on the process we developed for identifying policy-relevant landscape indicators, and the application of that process to the BLM. We then describe methods, results, and agency feedback on the four pilot applications. Finally, we discuss our process, datasets, and methods along with the importance of science-management partnerships in efforts such as this one that attempts to bridge the gap between science and its use in public lands decision-making. We close by identifying specific ways in which these indicators can be used to inform future planning and management decisions on multiple-use public lands.

## Identifying Policy-Relevant Landscape Indicators

**Fig. 1:**
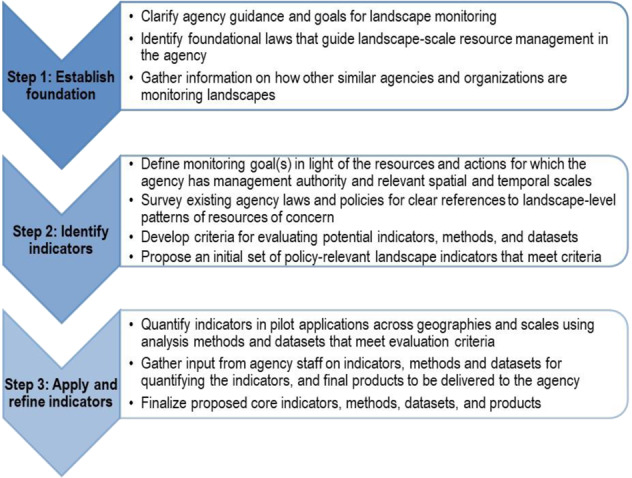
Key steps for identifying policy-relevant landscape indicators, methods, and datasets for public land management agencies

We began our effort to identify core landscape indicators by holding a scoping workshop with a group of 42 science and technical advisors to generate an initial outline of a process to identify the indicators, and then followed up with participants and other agency staff to solidify the key steps, information needs, and desired products for public land managers. This process was modeled after those used previously by the BLM to identify core indicators for field-based monitoring of terrestrial and lotic systems (MacKinnon et al. 2011; BLM 2015a). The advisors included academic and federal researchers, land managers, and other personnel directly involved in resource monitoring for the BLM, US Geological Survey (USGS), US Forest Service, and National Park Service. Advisors provided expertise in (1) environmental monitoring, particularly at broad spatial scales and across ecosystems, (2) methods and datasets useful for quantifying ecological patterns and processes, and (3) agency management, planning, and policy making. To help ensure that the process satisfied the practical needs of a federal land-management agency, we (USGS and BLM) adopted a coproduction approach (e.g., Meadow et al. 2015; Beier et al. 2017) to the effort, including having multiple BLM staff on the team responsible for leading and conducting the project and talking frequently with policy and management staff at BLM field, state, and national offices. The resulting process is organized as three sequential steps (Fig. 1).

### A Process for Identifying Policy-relevant Landscape Indicators

Step 1 establishes a foundational basis for monitoring landscapes (Fig. 1) by examining policy, laws, and regulations that guide agency actions. Gathering information on efforts by other agencies and organizations with similar missions and management responsibilities to assess and monitor landscapes can help an agency determine what approaches may be best suited to its own needs and facilitate adoption of aspects of existing programs or approaches that can promote cross-agency use of the resulting data (Toevs et al. 2011b).

In step 2, an initial set of indicators is proposed that align with the agency’s monitoring goal(s). Documenting clear references to landscape-level resource management goals and reporting needs in existing agency laws and policies is critical for identifying indicators that will be relevant to agency work. Indicators that can inform or facilitate required reporting are most likely to be viewed as useful by agency staff.

Step 3 is to quantify the proposed indicators in pilot applications and gather input from agency staff to help ensure that the indicators are feasible to quantify, clearly presented to staff in easy to use formats and products, and relevant to agency planning and management across different geographies, ecosystems, and management contexts. Steps 2 and 3 are both likely to be iterative.

### Application of the Process to BLM

Two foundations for the effort were articulated by our project advisors. First, because many changes in public lands policy were occurring at the time, advisors emphasized that the indicators needed to be grounded in foundational laws that are unlikely to be altered as elected officials and their administrations change over time. Accordingly, we identified the Federal Land Policy and Management Act of 1976, the Fundamentals of Rangeland Health, and the National Environmental Policy Act [USC §4321] as foundational to BLM resource management.

Second, advisors recommended eight guiding principles for the effort which focused on relevance to BLM reporting and simplicity in terms of concept and interpretability (Table 1). The advisors emphasized the need for an inclusive, transparent, science-based process that would build agency understanding and buy-in for implementation, and indicators that would be feasible to assess (and eventually monitor over time) using existing data, technologies, and resources. BLM also required that the effort be consistent with principles in BLM’s assessment, inventory, and monitoring (AIM) Strategy (Toevs et al. 2011a, 2011b; Taylor et al. 2014) and consider information and lessons learned from a suite of rapid assessments that quantified the amount and distribution of vegetation types, and some connectivity and patch-based metrics, at ecoregional scales across much of the western US (e.g., Comer et al. 2013; Carr and Melcher 2017; Wood et al. 2016).

**Table 1:** Key guidance and recommendations for identifying landscape indicators provided by science and technical advisors from within and outside of the Bureau of Land Management

1. Articulate a clear, policy-based explanation for why the agency needs to conduct landscape-level monitoring.
2. Ensure that the spatial pattern information is directly relevant to foundational agency laws and policies and helpful for guiding agency management decisions (e.g., land use planning, permitting for grazing, and oil and gas development).
3. Focus on indicators that provide information on spatial patterning of resources and landscapes that will be complementary to ongoing field-based monitoring programs.
4. Clearly connect landscape indicators to real management questions that reflect major current resource management challenges for the agency.
5. Define terms clearly, as landscape indicators will be a new concept to many managers.
6. Consider indicators that can foster a collaborative, all-lands approach to management, and that may already be monitored through other agency or interagency efforts.
7. Explain how the indicators can be used to inform local, project-level decisions, to help understand mechanisms and drivers of change in resource condition, and to help build manager interest and buy-in over the long term, as managers have struggled to see the relevance of landscape metrics to their day-to-day work in previous landscape-scale assessment efforts.
8. Embrace the opportunity to develop consistent, standardized methodologies from the outset for landscape indicators while acknowledging that identifying indicators for monitoring landscape patterns may be more difficult because of the relative newness of the science.

We also explored if and how other organizations are assessing and monitoring natural resources at landscape scales. A few clear patterns and lessons emerged. First, multiple organizations have taken steps toward landscape-level assessment and monitoring of vegetation patterns, including the Environmental Protection Agency (Environmental Protection Agency 1994, 2002), National Park Service (Monahan et al. 2012), and US Forest Service (Wurtzebach et al. 2019). Second, all of these monitoring efforts start from a foundation of monitoring resource structure, composition, and function (Noss 1990). This foundation leads to indicators that quantify the amount, mapped distribution, and metrics that describe spatial pattern (e.g., patch size, connectivity, fragmentation) at one or more levels in the ecological hierarchy (Noss 1990). Third, organizations are monitoring, or acknowledge the need to monitor, metrics of both resource patterns and of stressors potentially affecting those resources (e.g., fire, development, invasive species) to better understand relationships across broad areas as they relate to management activities. Taken together, it was apparent that organizations see the need for landscape-level monitoring information that managers can easily use as part of their decision-making processes, but that a consistent and well-accepted set of landscape indicators that managers across agencies use to inform their planning and management actions is lacking.

Based on our findings from Step 1, we revised our goal for landscape monitoring in the BLM to a more feasible goal of quantifying the current status of patterns of vegetation types of management concern at scales relevant to agency actions (e.g., grazing allotments, field offices, states) using datasets likely to provide informative results over timeframes coinciding with required land use plan reporting (5–10 years).

We identified BLM land health standards and indicators as the key mechanisms most directly connecting BLM policy to landscape concepts. BLM land health standards are derived from the Fundamentals of Rangeland Health (43 CFR §4180): (1) watersheds in properly functioning condition, (2) ecological processes that support healthy biotic populations and communities; (3) water quality that complies with state standards and achieves BLM objectives; and (4) habitats that support special status species. From these fundamentals, the BLM worked with 19 Resource Advisory Committees to develop land health standards and indicators for different geographic areas (Kachergis et al. 2020), which figure prominently in BLM land use plans and guide agency management and restoration actions. We examined all land health standards and indicators to determine how frequently landscape concepts (i.e., potential landscape indicators) were referenced, and the resources (e.g., soil, vegetation, surface water) to which the indicators referred (Table 2).

**Table 2:** Prevalence of potential landscape indicators and the resources they refer to in Bureau of Land Management (BLM) land health standards across 19 BLM Resource Advisory Committee areas

Resource advisory committee area	Amount and distribution	Diversity	Patch size	Connectivity/corridors	Fragmentation
Alaska	G, H, O	G, S		V/H	O
Arizona	G, O	S			
California (NW)	G, S, H, O	S	V/H	V/H	O
California (NE) and Nevada (NW)	G, S, H, O	S	V/H	V/H	
California (Central)	G, S, H, O	S	V/H	V/H	O
California Desert District	S	S			
Colorado	G, S, O	G, S		V/H	H
Idaho	G, S, H, O	G, S			
Montana (Butte, Dillon, Missoula FOs)	G, O	G, S		V/H	H
Montana (Lewistown and Malta FOs)	G, S, O	G, S		V/H	H
Montana (Miles City and Billings FOs)	G, S, O	G, S		V/H	H
North and South Dakota	G, S, O	G, S		V/H	H
Nevada (Mojave-Southern Great Basin)	G, S, H, O	G, S	V/H	V/H	
Nevada (Sierra Front - NW Great Basin)	G, S, O	G, S	V/H	V/H	H
Nevada (NE Great Basin)	G, S, H, O	S	V/H	V/H	
New Mexico	G, S, O	G, S			
Oregon and Washington	G, H, O	S		V/H	O
Utah	G, S, H, O	S		V/H	
Wyoming	G, S, H, O	S			H
Total Resource Advisory Committee areas	G: 18, S: 15, H: 10, O: 18	G: 10, S: 19	V/H: 6	V/H: 12	H: 7, O: 4

Letters refer to the resource addressed: G: vegetation generally, vegetation types, or vegetation communities; S: individual species or groups of species of vegetation, H: habitats (e.g., special status species), O: other (e.g., litter, biological soil crusts, rock, bare ground), V/H: vegetation or habitats

We then agreed upon eight criteria that potential indicators, methods, and datasets would need to satisfy (Table 3). Evaluation criteria addressed relevance to specific reporting requirements and the scientific foundation, variability, and quality of available data to derive metrics. Of the indicators frequently referenced in BLM land health standards and indicators, five were referenced, including in landscape contexts, across a majority of BLM land health standards and indicators, were consistent with input from our science and technical advisors, and met the evaluation criteria: amount, distribution, patch size, structural connectivity, and diversity of vegetation types. These became our proposed core landscape indicators. Many other combinations of indicators and resources were suggested and considered in the initial workshop or during our review of BLM land health standards and indicators (e.g., number of dams per stream, fragmentation of biological soil crusts), but did not meet our criteria.

**Table 3:** Criteria for evaluating potential indicators, datasets and methods to quantify landscape-level patterns of ecological resources to inform agency management decisions

Criteria	Description
Landscape relevance	1. Indicator quantifies the amount or spatial pattern of an ecological resource.
Policy relevance	2. Indicators can be used to assess compliance with foundational laws and policies relevant to agency management.
Spatial relevance	3. Indicator is relevant and can be quantified using available datasets, across lands managed by the agency and its partners.
Interpretability and usability	4. Indicator is responsive to disturbances or management actions on time scales relevant to major management decisions (e.g., 5–15 years).
	5. Indicators can be used by managers to identify goals and set quantitative objectives in land use plans and other decision documents.
	6. Quantitative reference or desired conditions for the indicator are feasible to identify.
Scientific foundation	7. Indicator is well documented in the peer-reviewed scientific literature as useful for landscape-level assessment, inventory, and monitoring.
Compatibility	8. Indicator, analysis methods, and source datasets are consistent with, compatible with, or informed by those currently used by the agency and its management partners.
Response variability	9. Environmental factors controlling the natural temporal and spatial variability of the indicator are well understood.
Data quality and feasibility of implementation	10. Indicators can be quantified using widely accepted and used datasets with complete coverage across the western US (including Alaska) that are of consistent quality and are regularly updated.
	11. Indicators can be quantified using well-accepted and documented analysis methods in a minimal number of steps.
	12. Indicator results are likely to be of sufficient quality (i.e., within acceptable error/uncertainty tolerances) to be scientifically credible and useful for management.
	13. Indicators can be quantified using existing datasets with acceptable accuracy and precision.
	14. Time and cost needed to quantify the indicator across spatial extents relevant to agency planning and management actions are reasonable.

We describe the below results from Step 3, which is to quantify the proposed indicators in multiple locations, gather feedback from agency staff on their utility, and finalize a recommended set of indicators, methods, and datasets.

## Pilot Applications

### Study Areas

**Fig. 2:**
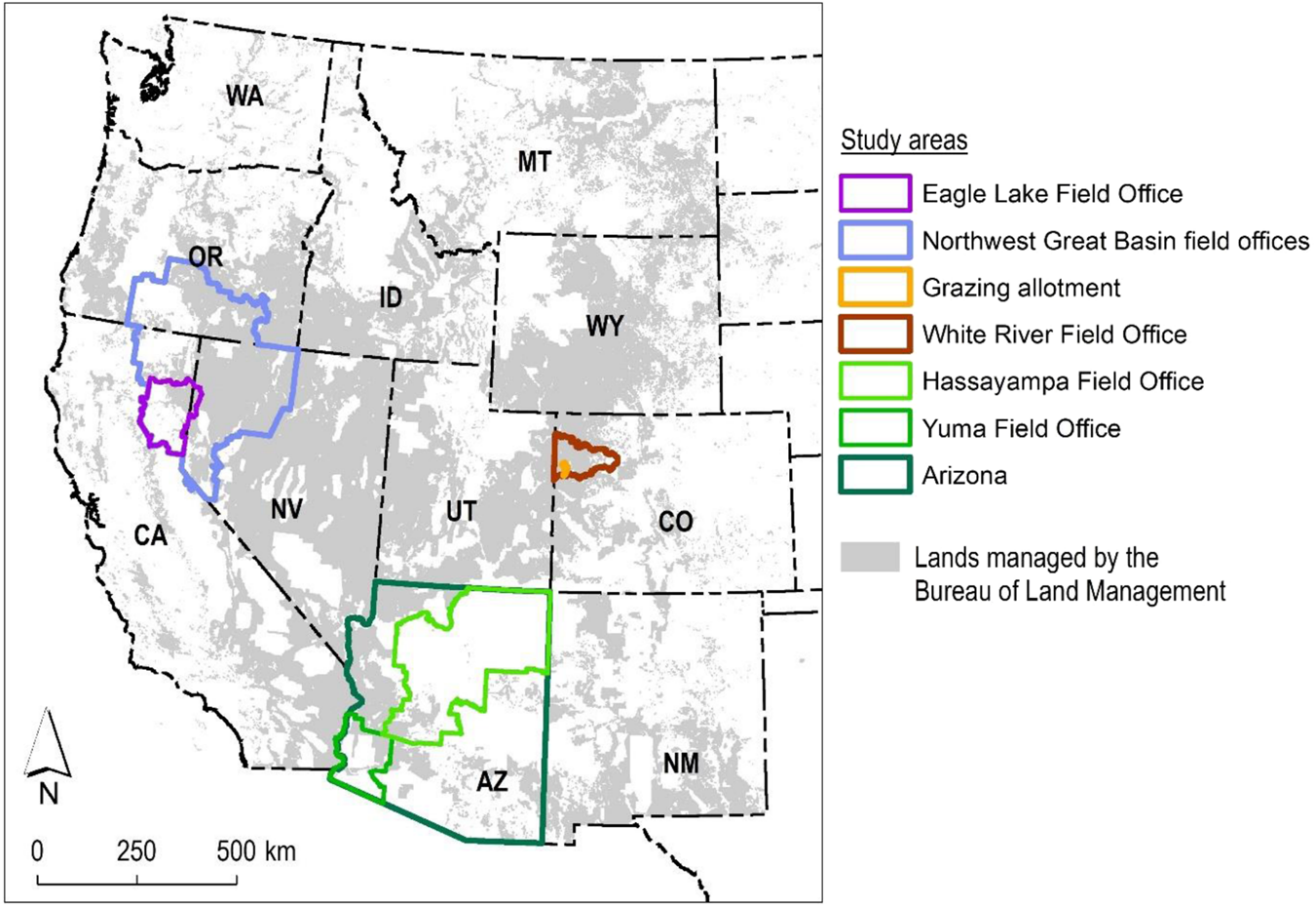
Study area boundaries for the four pilot applications conducted in the Bureau of Land Management (BLM) field offices in Arizona, California, and Colorado to refine procedures for quantifying landscape indicators and assess the utility of those indicators for managers. For each boundary, landscape indicators were quantified for both BLM-managed lands and for non-BLM lands

We chose four BLM field offices in Arizona, California, and Colorado to test the proposed indicators (Fig. 2). Each pilot application quantified the indicators within two boundaries relevant to current plans or management issues: a focal boundary and a relevant larger boundary to provide context for interpreting results from the focus area. The Yuma and Hassayampa Field Office pilot applications both used the state of Arizona as the larger boundary. In Colorado, we chose a mid-sized grazing allotment as the focus area within the White River Field Office. In California, the Eagle Lake Field Office was the focal boundary within a larger area defined by the outer boundaries of eight field offices in the northwestern Great Basin. Boundary selection was guided by BLM planning documents, current management issues identified by BLM state and field office staff, and a desire to quantify indicators across a range of spatial extents.

## Methods

We used BLM resource management plans (with amendments), land health standards and indicators for the area, and input from field and state office staff (e.g., wildlife biologists, fire ecologists, geospatial ecologists, planners, monitoring coordinators) to identify priority vegetation types and relevant vegetation management objectives for each pilot application. BLM resource management plans are comprehensive documents that identify, among many other things, desired outcomes (goals and objectives) for vegetative resources, including desired mixes of vegetative types and structural stages; landscape and riparian functions; habitat for native plants, fish, and wildlife; and forage for livestock. Desired outcomes may be established at multiple scales. Plans may also identify areas of ecological importance, designate priority plant species and habitats, and identify actions and area-wide use restrictions needed to achieve desired vegetative conditions (BLM 2005).

We evaluated three datasets as sources for current vegetation: LANDFIRE Existing Vegetation Type (EVT 2.0, LANDFIRE 2019b), USGS GAP Landcover data (National Gap Analysis Program 2011), and the National Land Cover Database (Multi-Resolution Land Characteristics Consortium 2019). We chose LANDFIRE EVT as the basis for quantifying indicators because of its greater thematic detail for natural vegetation and its consistency in methodology across regions. We used LANDFIRE Biophysical Settings (BpS 2.0, LANDFIRE 2019a) to derive a metric related to estimated historical (pre-European settlement) vegetation types and BLM’s surface management agency dataset (BLM 2017) for land management boundaries.

We created linkage tables between LANDFIRE EVT and BpS class names using descriptions and species information in the NatureServe terrestrial ecological classifications (NatureServe 2018) and LANDFIRE Map Unit Descriptions (LANDFIRE 2016), along with information provided by Pat Comer (NatureServe, personal communications, August 2018 and October 2019). Priority vegetation communities identified in BLM resource management plans often consisted of multiple EVT classes. In those cases, we additionally used agency staff input to identify and produce 30-m raster maps of the EVT and BpS classes comprising the vegetation community. To be feasible for regular use by BLM, methods needed to be implemented using readily available software. We completed all analyses using ArcGIS 10.7 (ESRI 2019).

We quantified the indicators within both boundaries for each study area for both BLM and non-BLM lands for several reasons. The primary interest of BLM managers is the status of vegetation communities on lands that they manage in their field office. However, managers are also fully aware that BLM-managed lands occur within a matrix of other public and private lands which influence management options and outcomes across the entire landscape (Carter et al. 2020). Further, managers are interested in how vegetation patterns on their lands compare to those on lands managed by other entities and in the broader region surrounding their field office.

### Amount and Distribution

Using the EVT/vegetation community raster data, we derived the mapped amount of each vegetation type or community on BLM-managed lands and on all lands for each boundary and provided results for these and all metrics in a report format that could be easily inserted into agency planning documents.

### Patch Size and Connectivity

Many patch size and connectivity metrics exist (e.g., McGarigal et al. 2012), including methods and metrics for quantifying functional connectivity of habitats for individual species (e.g., Gray et al. 2019). For this study, our focus was on the structural connectivity of vegetation types across planning areas and larger landscapes. Our goal was to identify a simple metric that would encourage managers to begin considering and assessing the connectivity of vegetation types and communities quantitatively in their land use plans. Thus we chose two straightforward metrics—patch area and proximity to the nearest patch—for our pilot applications. We used two methods to create patches. We used a “contiguous” method for vegetation communities that tend to occur in larger, more numerous patches across the area of interest. This method required pixel adjacency to be considered part of the same patch and was implemented using the ArcGIS Spatial Analyst Region Group tool and an eight-neighbor adjacency rule. We used a “cluster” method for vegetation communities that tended to occur in small or linear patches (here, riparian and wetland vegetation). With this method, pixels separated by a minimum distance were considered part of the same patch. Here we defined a patch as all pixels of that vegetation community within 90 m of the focal pixel and created patches by buffering the pixels, dissolving the overlapping buffers, and assigning all pixels of that vegetation community within the dissolved buffer to the same patch. The 90 m threshold was derived from visual examination of imagery and the location of associated wetland/riparian pixels in the study boundaries across the three datasets, especially along the small streams that BLM typically manages. For both methods, we used the ArcGIS Generate Near Table tool to calculate the distance from each patch to the next nearest patch. We computed the frequency distribution of patch size and patch proximity by both percent of total area and percent of a total number of patches because of guidance from BLM staff that both were relevant to their decisions. When numerical management objectives for indicators existed, we graphed results using size and distance classes tailored to those objectives.

### Vegetation Type Diversity

To calculate vegetation type diversity, we used the ArcGIS Spatial Analyst Focal Statistics tool to determine the number of discrete EVT and BpS class names for natural vegetation types only (developed, agricultural, and other disturbed categories in EVT were excluded) within 500 m of a focal pixel. We first computed and mapped current vegetation type diversity in line with our monitoring objective to assess current status. Based on requests from BLM staff, we also computed and mapped the difference between current and estimated historic vegetation type diversity, using the difference between the count of class names for natural vegetation types between EVT and BpS for each neighborhood.

## Results

We first present results for the Yuma Field Office and then summarize results across the four study areas. Results for the Hassayampa, Eagle Lake, and White River field offices are provided in Appendices A, B, and C, respectively. For all study areas, we illustrate patch metrics for one common upland priority vegetation community and for riparian/wetland, a scarce lowland vegetation community that is always a priority for BLM managers.

### Priority Vegetation Communities and Management Objectives

The Yuma Field Office resource management plan (BLM 2010a) identifies seven priority vegetation types: riparian/wetland, mesquite bosques/woodlands, desert wash woodlands, paloverde mixed cactus, creosote white bursage, mountain uplands, and dune complexes. We were able to map four of these, and present results here for creosote white bursage and riparian/wetland.

Across our study areas, we identified between six and 12 priority vegetation communities based on the most recent resource management plans and amendments and contemporary issues noted by field staff. We were able to map and quantify the majority of those communities, but some were not well characterized by LANDFIRE EVT classes, and the area of a few communities was too small for meaningful analysis of landscape patterns. Notably, only one plan had a (partially) quantitative vegetation management objective related to landscape patterns. The Yuma plan included the objective: “VM-013—Manage for large, contiguous blocks of native riparian habitat (>30 acres) for yellow-billed cuckoo [*Coccyzus americanus*] in conjunction with removal of competing for exotic species (such as salt cedar)”. Our analysis can be used to partially evaluate this objective by considering the frequency distribution of patch sizes of all riparian/wetland vegetation.

### Patterns of Common Upland Vegetation Communities

**Fig. 3:**
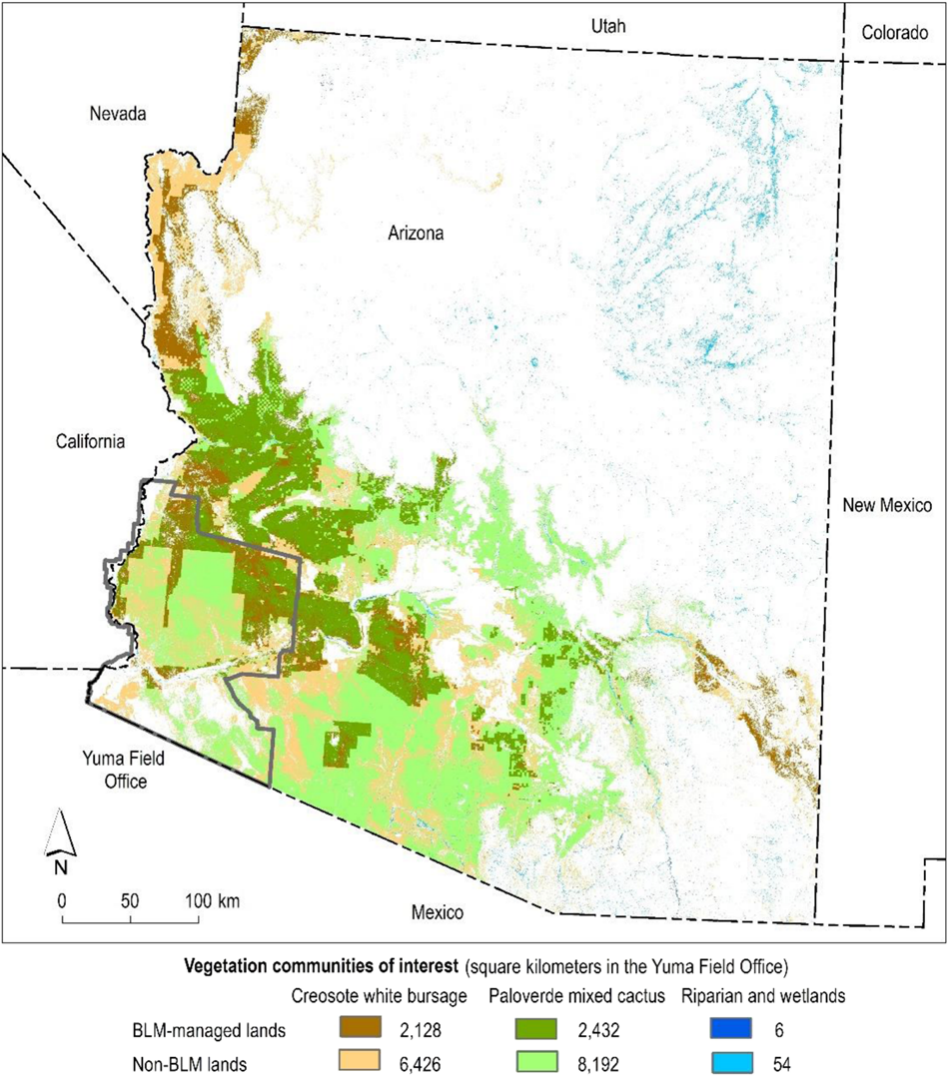
Priority vegetation types in the Yuma Field Office and the State of Arizona. Darker and lighter shades of each color represent the presence of the vegetation type on the Bureau of Land Management (BLM)-managed lands and on non-BLM lands, respectively

**Fig. 4:**
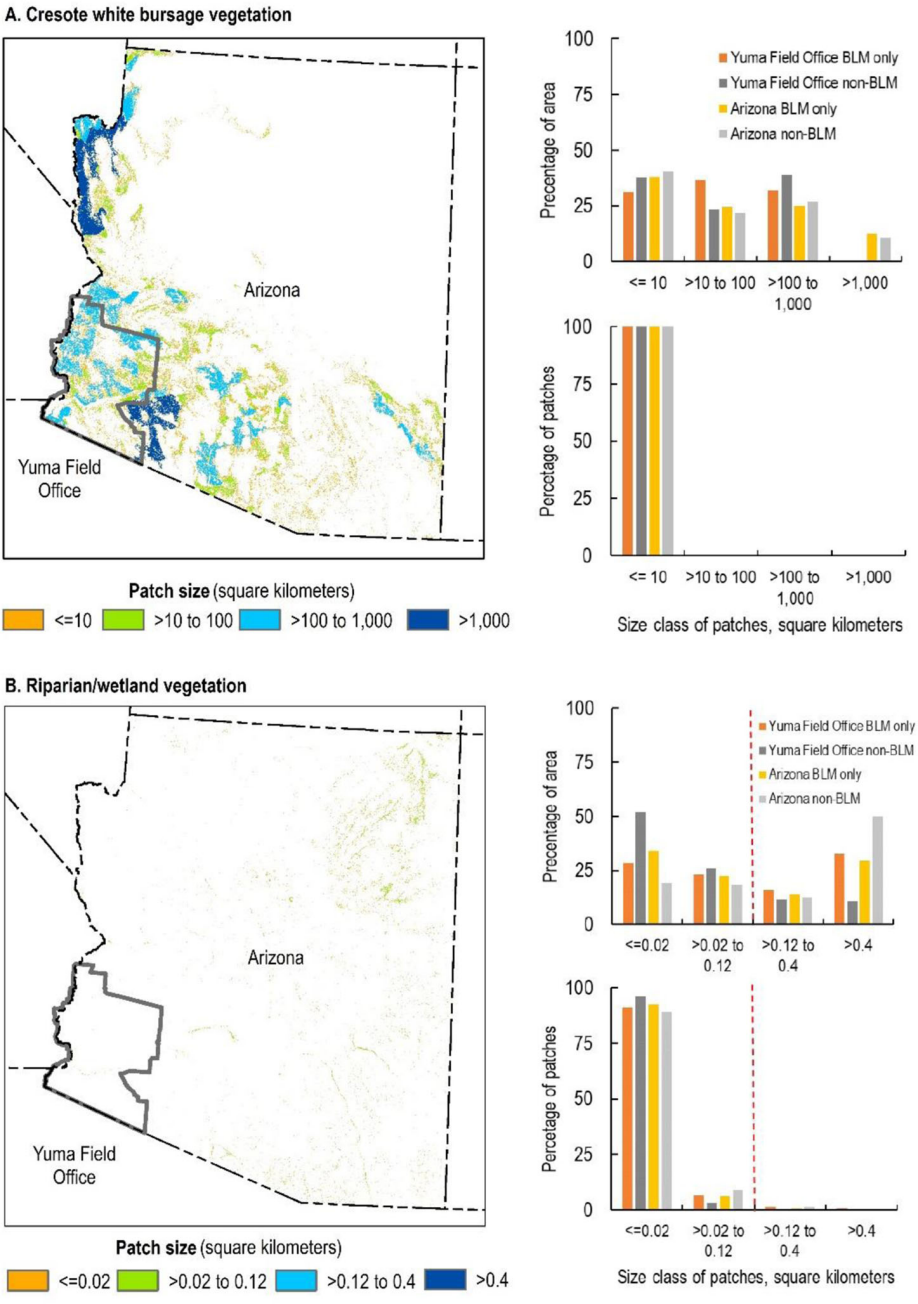
Patch sizes of creosote white bursage vegetation (**A**, top) and riparian/wetland vegetation (**B**, bottom) on lands managed by the Bureau of Land Management (BLM) and on non-BLM lands in the Yuma Field Office and Arizona. The red dashed line represents a measurable management objective for riparian/wetland vegetation patches in the Yuma Field Office Resource Management Plan (BLM 2010a)

**Fig. 5:**
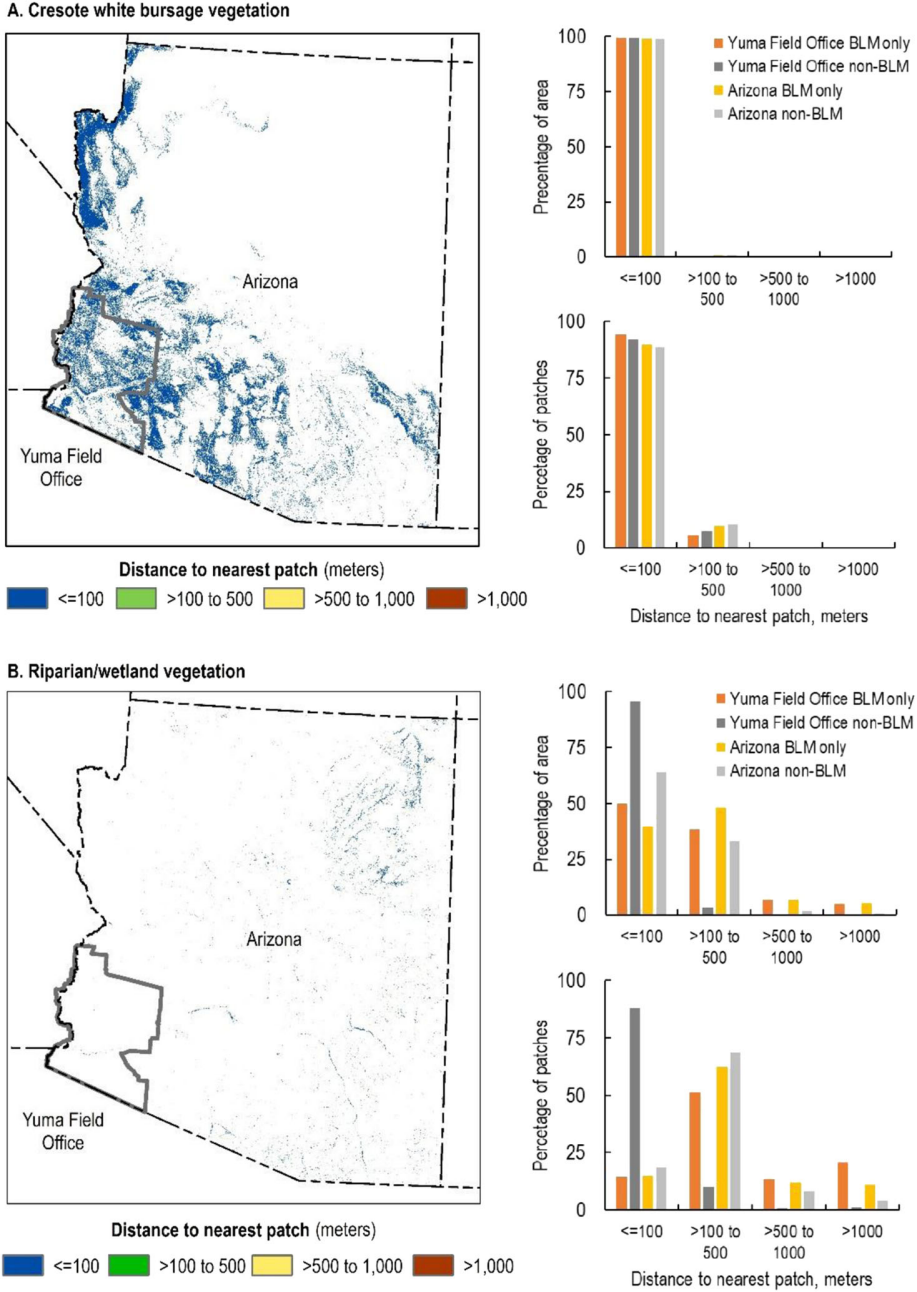
Patch proximity of creosote white bursage vegetation (**A**, top) and riparian/wetland vegetation (**B**, bottom) on the Bureau of Land Management (BLM)-managed lands and non-BLM lands in the Yuma Field Office and Arizona

Creosote white bursage is present on lands across the Yuma Field Office, comprising 43 and 28% of BLM and non-BLM lands, respectively (Fig. 3). The vast majority (99%) of patches are 10 km^2^ or smaller in size, however, the total area of creosote white bursage is relatively evenly distributed across three patch-size classes (≤10, 10–100, and 100–1000 km^2^) in both the field office and the state, with no clear difference between BLM and non-BLM lands (Fig. 4A). Nearly all (88.9%) patches occur within 100 m of the next nearest patch across scales and ownerships (Fig. 5A).

Across the study areas, common upland vegetation communities of management concern are often distributed across only a portion of the field office and fragmented by other land ownerships and jurisdictions (Figs. 3, A1, B1, C1). The total area of these vegetation types is often distributed among multiple patch size categories, with BLM tending to manage areas in larger patch size classes compared to non-BLM lands. For example, 59% of the area of Paloverde mixed cactus on BLM lands in the Hassayampa Field Office occurs in patches >1000 km^2^ compared to 28% for non-BLM lands (Fig. A2A). However, there are always far more small patches (<10 km^2^) in terms of absolute numbers (Figs. 4A, A2A, B2A, C2A). Patches of common upland vegetation types are largely clustered, evidenced by the preponderance of nearest neighbor distances of ≤100 m on both BLM and non-BLM lands (Figs. 5A, A3A, B3A, C3A).

### Patterns of Riparian/Wetland Vegetation

The Yuma Field Office has very little riparian/wetland vegetation (0.1% of BLM lands, 0.3% of non-BLM lands, Fig. 3). About half (49%) of the total area of riparian/wetland on BLM-managed lands in the field office meets the agency’s objective for occurring within patches ≥30 acres (0.12 km^2^) in size, but only 2% of individual riparian/wetland patches are this large (Fig. 4B). On non-BLM lands in the field office, 22% of the total area of riparian/wetland occurs in patches ≥30 acres, with 0.4% of patches being this large. Riparian/wetland vegetation nearly always occurs within 500 m of the next nearest patch in both the field office and state (≥97% of the area of riparian/wetland vegetation on BLM lands, 88% on non-BLM lands, Fig. 5B).

Across the study areas, BLM consistently manages only a small area of riparian/wetland vegetation compared to the amount of upland vegetation and manages a smaller proportion of riparian/wetland than non-BLM entities (Figs. 3, A1, B1, C1). The total area of riparian/wetland vegetation tends to be evenly distributed among different patch size categories, although there is notably more riparian/wetland vegetation in larger patches (>0.4 km^2^) in the Eagle Lake Field Office and northwest Great Basin on both BLM lands (73 and 87%, respectively) and non-BLM lands (60 and 69%, respectively, Fig. B2B). However, there are always far more very small patches (≤0.02 km^2^) of riparian/wetland vegetation in terms of absolute numbers (Figs. 4B, A2B, B2B, C2B). Most riparian/wetland patches are 100–500 m from their next nearest neighbor on both BLM and non-BLM lands at all extents (Figs. 5B, A3B, B3B, C3B).

### Patterns of Vegetation Type Diversity

**Fig. 6:**
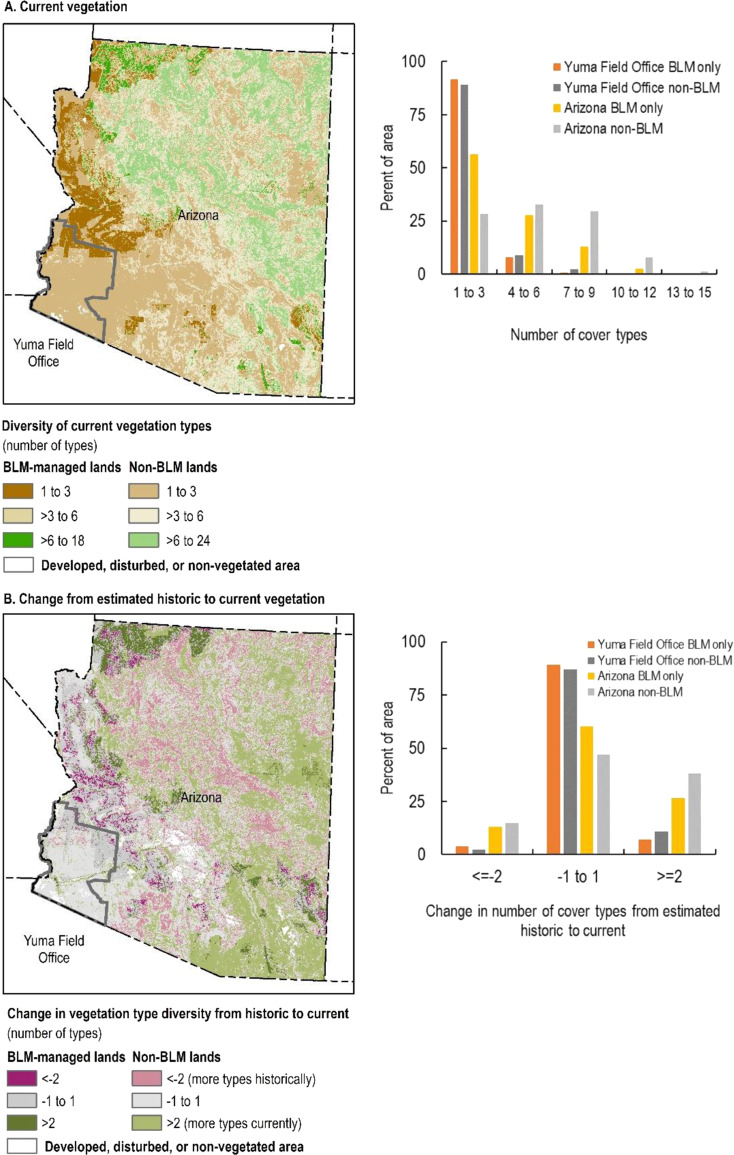
Diversity of current natural vegetation types (**A**) and change in the diversity of natural vegetation types between estimated historic (pre-European settlement) and current vegetation (**B**) in the Yuma Field Office and Arizona. Darker and lighter shades of each color represent Bureau of Land Management (BLM)-managed lands and non-BLM lands, respectively

Vegetation type diversity is lower in southwest Arizona where the Yuma Field Office is located compared to the rest of the state (Fig. 6A). In Arizona overall, BLM manages more lands of lower vegetation type diversity than non-BLM entities (57% of BLM lands have 1–3 natural vegetation cover types in the surrounding landscape, compared to 28% for non-BLM lands). Most lands in the field office and state did not show a substantial decrease in the number of natural vegetation types compared to estimated historic conditions (4 and 2% of BLM and non-BLM lands, respectively, in Yuma, showed a decrease of ≥2 natural vegetation types; 13 and 15% of BLM and non-BLM lands, respectively, showed this level of decrease in Arizona, Fig. 6B).

Patterns of diversity vary across the four study areas, with the two field offices in Arizona tending to manage lower diversity lands (1–3 vegetation types within 500 m), while the BLM field offices in California and Colorado manage lands across a range of diversities (Figs. 6A, A4A, B4A, C4A). BLM tends to manage lands of lower vegetation type diversity compared to non-BLM entities in all study areas. Across field offices, few lands (both BLM and non-BLM) showed a decrease in diversity of natural vegetation types from estimated historic to present (Figs. 6B, A4B, B4B, C4B). For example, only 4 and 8% of BLM and non-BLM lands respectively, in the Eagle Lake Field Office, and 6 and 11% of BLM and non-BLM lands, respectively, in the northwest Great Basin showed a decrease in cover type diversity of 2 or more natural vegetation types (Fig. B4B).

### Manager Perceptions of the Utility of the Indicators and Results Across Study Areas

As part of the pilot applications (i.e., Step 3), we worked closely with BLM state and field office staff and gathered informal feedback from them on both the indicators and the quantitative results of our analyses in their region over the course of multiple meetings and work sessions. BLM staff across levels and positions appreciated the baseline information provided by the indicators for their priority vegetation communities and considered it to be directly relevant to (and in a format that can now be easily integrated into) their land use plans. The indicators afford them a better understanding of the current status of their resources, which they can use to identify vegetation treatment and restoration needs. For example, the limited amount of riparian/wetland vegetation managed by the BLM was expected based on patterns of settlement in the western US in which lands along streams were primarily secured by settlers (e.g., Sauder 1989), but our analysis provided managers with an estimate of the area of riparian/wetland vegetation that satisfies the specific patch-size objective in the Yuma Field Office. Staff indicated that they can use this estimate along with the mapped patch sizes to determine the need to manage for more ≥30-acre patches, and to target management actions where they have the best chance of achieving and sustaining this objective (e.g., expanding or connecting small riparian/wetland patches that are surrounded by other natural vegetation). Managers also noted the utility of the indicators for responding to national and state-level data requests (e.g., about the status of BLM-managed riparian areas) and for informing management decisions (e.g., renewal of livestock grazing permits). BLM staff may also use the indicators for identifying areas of high vegetation type diversity across landscapes that may warrant additional protection or for targeting field sampling (e.g., to measure plant species diversity). Staff wanted to extend the use of the indicators beyond an assessment of current status to quantifying recent trends, comparisons with historic conditions, and relationships with potential drivers of change. Staff also wanted to use this landscape-level information together with field-based vegetation data to better map key plant communities that are poorly mapped by LANDFIRE EVT (e.g., bitterbrush (*Purshia tridentata*) in the northwestern Great Basin). Staff were concerned about the accuracy of vegetation data in their field office and identified this as a major challenge to broader implementation and regular use of landscape metrics in decision-making.

## Discussion

We took an initial step in bridging the gap between landscape science and public land management (Carter et al. 2020) by identifying, from among the many existing landscape concepts and metrics, a small suite of core indicators that relate directly to existing policy and plans for federally managed multiple-use public lands in the US. We developed a process for identifying such indicators and recommend that the amount, distribution, patch size, structural connectivity, and diversity of natural vegetation types be an initial set of landscape indicators for assessing vegetation patterns to inform BLM management of public lands. As a proof of concept, we quantified the indicators in four BLM field offices across the western US. The indicators that resonated with agency planners, managers, and policy makers, were relevant to their management issues and decisions and were feasible to assess using existing data and technologies.

### The Role of Science-management Partnerships in Bridging the Landscape Science-management Gap

The value of this effort was not in identifying complex, state-of-the-art landscape metrics. There are already many of these (e.g., McGarigal et al. 2012; Vogt and Ritters 2017). Instead, we sought to identify straightforward metrics that public land managers would actually use in their planning and management decisions. To do this, we relied extensively on a collegial coproduction partnership (Meadow et al. 2015) between the USGS and BLM. Staff from both agencies jointly developed the process for identifying indicators, applied that process in multiple BLM field offices, and regularly discussed ideas and gathered feedback from agency staff on the usefulness and specific potential uses of the indicators and metrics. BLM state and field office staff helped identify priority vegetation communities and provided input on datasets and rules for mapping vegetation communities using EVT classes in LANDFIRE. Patch definitions for rare or linear vegetation types were also developed in concert with managers and in response to observed limitations of LANDFIRE in mapping these vegetation types in their field offices. Agency staff also provided input on product content and formats for presenting indicator results that they could easily integrate into their planning and decision documents. Coproduction approaches such as these tend to produce science products at scales useful for managers and that are more easily integrated into agency decision-making processes (Meadow et al. 2015).

### The Process for Identifying Indicators

Traditional, formal methods for identifying ecological indicators for monitoring employ a systems-like approach using conceptual models of varying detail to highlight key ecosystem drivers and their effects on biophysical components (Miller et al. 2010; Miller 2005; O’Dell et al. 2005). Our approach differed from tradition in that the knowledge base of science and technical advisors, agency foundational laws, and a detailed understanding of agency workflows supplanted the direct use of conceptual models to guide the selection of indicators. Although we primarily used agency land health standards (Table 2) and criteria and guidance derived from the literature and advisors’ recommendations (Tables 1 and 3) to guide the selection of landscape indicators, we purposely limited the initial number of indicators to better achieve our goals of feasibility and eventual use by public land managers. Additional indicators or metrics will be needed for targeted monitoring, such as monitoring habitat for individual wildlife species of management concern (Toevs et al. 2011a, 2011b; Taylor et al. 2014).

We emphasize that these indicators and analyses for priority vegetation types are only one part of the information needed to inform landscape-level decisions on multiple-use public lands. Data on additional resources of management concern (e.g., soils, surface and ground water) and on anthropogenic and ecological processes that affect resources (e.g., climate, fire, development, invasive species, disease, Wood et al. 2016; Carr et al. 2016) are also critical. In addition, using indicators of spatial pattern quantified across landscapes together with field-based monitoring metrics that are collected locally has the potential to compensate for the limitations of each: field-based measures lack continuous, spatial pattern information while landscape indicators lack detailed species-level information. A principle of BLM’s AIM strategy is the complementary integration of landscape and field-based measures to enhance the spatial and thematic detail of mapped resources to inform management (Toevs et al. 2011a, 2011b; Taylor et al. 2014). Current data integration efforts use field-based AIM data for training and accuracy assessment of remotely sensed products (e.g., Jones et al. 2018; Rigge et al. 2020; Zhou et al. 2020) and to improve mapping of high priority vegetation species (e.g., Young et al. 2020). Using analogous approaches that integrate agency field data with the proposed indicators may increase their utility.

### Datasets for Quantifying the Landscape Indicators

The importance of manager trust in the landcover data underlying landscape pattern analyses cannot be overstated, and the landcover data we used to quantify the indicators are not without limitations. There are challenges with LANDFIRE EVT accurately classifying vegetation types that tend to occur in very dry or wet areas, as small or indistinct patches that grade gradually into neighboring vegetation types, or as narrow linear configurations (e.g., riparian areas). Many vegetation types that are priorities in land use plans are priorities because they are a rare or minor component of the landscape. One goal of the comprehensive remap of LANDFIRE EVT, which resulted in LANDIRE 2.0, was to increase classification accuracy for some of these vegetation types, including riparian (Jim Smith, The Nature Conservancy, personal communication (oral), November 2018). The large units used for assessing the accuracy of LANDFIRE EVT, while ecologically based and appropriate for a nationwide dataset and the limited number of available testing data for each vegetation type, are part of this challenge. Agency land managers would be more convinced of the quality of the data if accuracy was assessed at the smaller extents (states, field offices) at which they are likely to use the indicators. Another federal land management agency in the US, the National Park Service, decided to use a different landcover dataset (the National Land Cover Dataset, e.g., Multi-Resolution Land Characteristics Consortium 2019) to quantify landscape patterns (DeVivo et al. 2018). This dataset has limited thematic resolution, which was not acceptable to BLM staff, but has the benefit of consistency in methodology and over time and relatively regular updates.

Consistent, westwide coverage of landcover data was a requirement for this project. However, it may be helpful in some cases to use specialty or locally available datasets with greater accuracy or thematic resolution for a specific area or vegetation community. For example, the National Wetlands Inventory (U.S. Fish and Wildlife Service 2018) may provide more detailed information on the distribution of wetland or riparian sites in some locations. Given the ultimate goal of not just assessing but of monitoring changes in vegetation patterns over land use planning timeframes (e.g., 5–15 years), any specialized or localized datasets would ideally be derived using consistent, well documented, repeatable methods and have a history and forecast of regular, comprehensive updates. Because LANDFIRE EVT 2.0 was produced using different methods than previous versions, it is not clear whether successive versions of LANDFIRE will provide the consistency in modeling and classification methods needed to enable its future use for monitoring change in vegetation patterns over time.

### Methods for Quantifying the Landscape Indicators

Our methods for quantifying the indicators were intentionally straightforward to foster a clear understanding of the results by managers and stakeholders, and to facilitate the ultimate calculation of metrics via desktop or web-based tools that may be used across very large extents (e.g., states, regions). We suggest that the methods and specific metrics used here to be viewed as a starting point. We hope that as managers become familiar and comfortable with these landscape concepts, they will want additional and more nuanced information about the landscape-level patterns of the resources they manage. Connectivity, in particular, is an area of focus currently among both federal and state resource managers (US Department of the Interior 2017c, 2018b; BLM 2018, 2019b). Connectivity metrics based on proximity, graph theory, network theory, and circuit theory each have different strengths, weaknesses, and utility for informing conservation and management actions (e.g., Baker et al. 2015, Rayfield et al. 2011, Simpkins et al. 2018). Managers may want to use a metric that better captures overall connectivity patterns or incorporate complementary analyses on corridors or functional connectivity for key plant or wildlife species. Similarly, managers may desire that the indicators be quantified for aspects of vegetation condition such as productivity (e.g., using eMODIS Remote Sensing Phenology, Jenkerson et al. 2010).

### Using the Landscape Indicators to Inform Public Land Management

Our goal was to identify indicators relevant to public lands policy and demonstrate their relevance to and feasibility for use in public land management. We believe involvement and input from a broad group of stakeholders and agency staff throughout this project were a core strength of the process we developed to identify policy-relevant landscape indicators. We have highlighted above a number of challenges to their regular use, including the availability of landcover datasets with an adequate thematic resolution that is produced at regular intervals using consistent methods. Limited staff time and agency expertise in landscape ecology can also hinder the use of landscape information in public lands decisions, as can the short timeframes in which many decisions must be made (e.g., Trammell et al. 2018, Cvitanovic et al. 2014). We suggest two specific future opportunities that could further use the indicators in public land management contexts, in addition to those mentioned above. First, adoption of the indicators by a resource management agency could be accompanied by the provision of reports and datasets for the quantified indicators to a broad suite of field offices. A subsequent survey of staff in those offices could help scientists and agency staff better understand how, and how often, field staff was able to use the indicators in their planning or management decisions as well as any barriers to use. Second, the landscape indicators were developed and calculated in a manner that lends itself to hypothesis testing and associated statistical analysis whenever resource managers think such analyses would be informative and helpful. For example, if BLM has set an objective for managing a particular vegetation type for large patch sizes (as is the case for riparian/wetland vegetation in the BLM Yuma Field Office in Arizona), quantifying the size distribution of patches over time using updated landcover data as part of periodic agency reviews of the effectiveness of land use plans can help managers determine if conditions on the ground are moving closer to meeting their vegetation objectives.

We suggest that these core landscape indicators can help to improve understanding, management of, and reporting on the health of multiple-use public lands in five main ways. First, the indicators provide a viable method for BLM to quantify existing landscape-level land health standards and indicators. BLM is required to manage public lands to meet these land health standards and recommends that land health be assessed at broad scales (e.g., watersheds, BLM 2009), but there has been no standard method for agency planners and managers to quantify these patterns. As a result, landscape aspects of land health standards have largely gone unmeasured. Second, many land use plans for public lands include landscape-level concepts and objectives for managing vegetation (e.g., Trammell et al 2018). These objectives, however, are nearly always qualitative. For example, the Bradshaw Harquahala Resource Management Plan for the Hassayampa Field Office in Arizona has a vegetation management objective to ‘maintain, restore, or enhance the diversity, distribution, and viability of populations of native plants, and maintain, restore, or enhance overall ecosystem health’ (BLM 2010b). Our indicators can provide current, baseline information that managers can use to set quantitative vegetation management objectives in future plans. Third, for agencies, including the BLM and US Forest Service, that require reporting on the effectiveness of land use plans (e.g., BLM 2005), our indicators provide a mechanism for evaluating progress in meeting landscape-scale management objectives at the spatial scales at which planning occurs (typically BLM field offices, see suggested example in the previous paragraph). Land use plan evaluations in the BLM currently focus on qualitative measures related to planning implementation, missing an opportunity to assess how implementation actions may have altered the landscape. Fourth, the National Environmental Policy Act (NEPA, 40 CFR §1500 et seq.) is a foundational environmental law in the US, guiding the decision process for many BLM planning and management actions. NEPA requires that federal agencies evaluate the potential effects of proposed actions on public lands. Recent (2020) changes to the NEPA regulations have altered the language used to describe such effects, but have not altered the requirement for federal agencies to fully analyze potential effects of proposed actions (see also US Department of the Interior 2021). Large projects have the potential to affect resource patterns at landscape scales. Landscape indicators provide one mechanism for standardizing how the full suite of potential effects—direct, indirect, and cumulative (Council on Environmental Quality 1997, BLM 2008b)—may be addressed and quantified in agency NEPA analyses. Finally, the US Department of the Interior has committed to managing across landscapes (US Department of the Interior 2017b). Measuring a core set of indicators using consistent methods and datasets across the western US can facilitate efforts to manage natural resources at landscape levels and provide a foundation for an all-lands approach to the management of multiple-use western landscapes.

## Supplementary information

Supplementary Information
