# Supplementary material for: Identifying Policy-relevant Indicators for Assessing Landscape Vegetation Patterns to Inform Planning and Management on Multiple-use Public Lands
Source: Environ Manage. 2021 Jun 26;68(3):426–43. doi: 10.1007/s00267-021-01493-8 (PMC8384788; doi:10.1007/s00267-021-01493-8)

**Supplementary Information**

**Appendix A: Hassayampa Field Office, Arizona.**

Figure A1. Priority vegetation types in the Hassayampa Field Office and State of Arizona. Darker and lighter shades of each color represent presence of the vegetation type on Bureau of Land Management (BLM)-managed lands and on non-BLM lands, respectively.


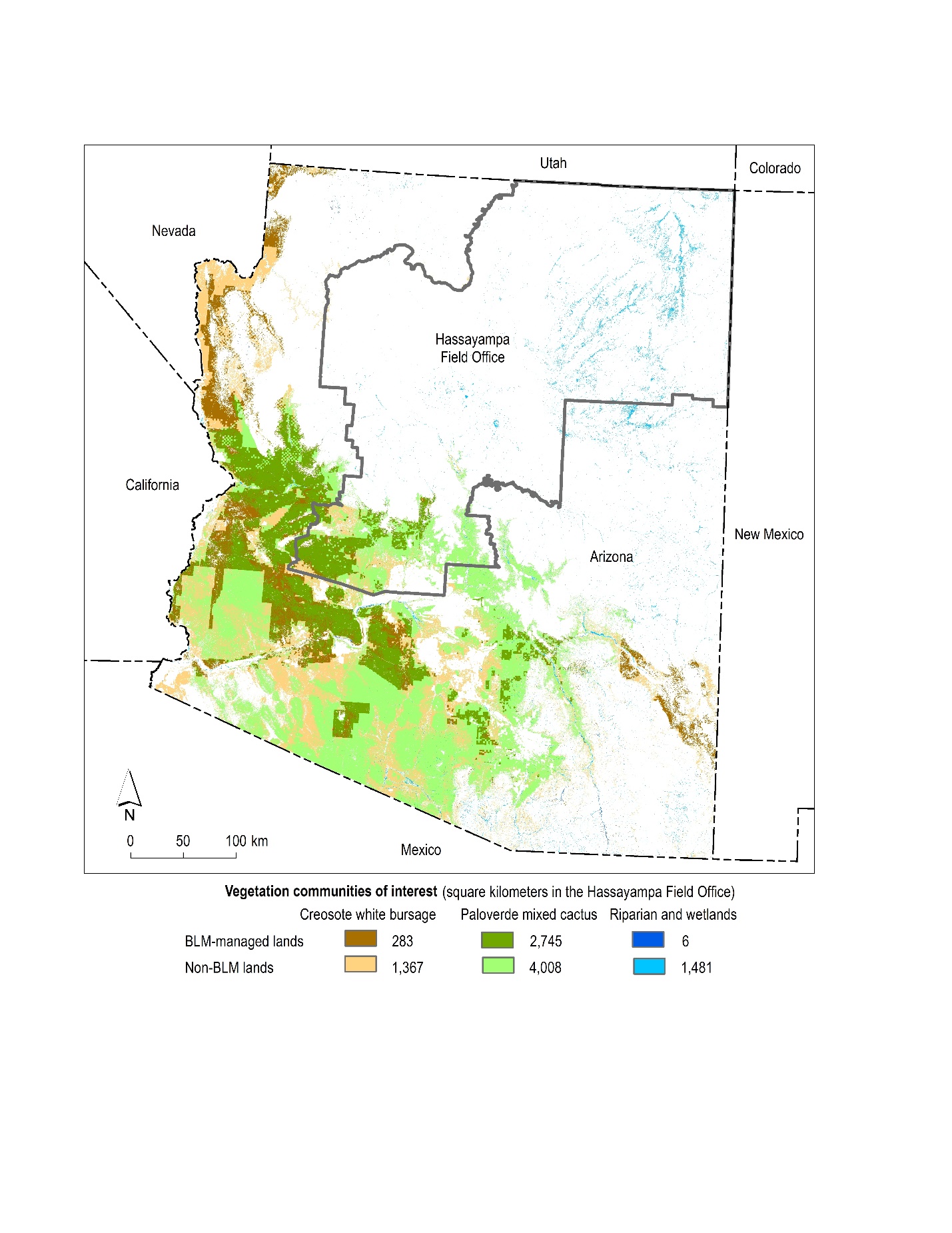


Figure A2. Patch sizes of paloverde mixed cactus vegetation (A, top) and riparian/wetland vegetation (B, bottom) on Bureau of Land Management (BLM)-managed lands and on non-BLM lands in the Hassayampa Field Office and Arizona.


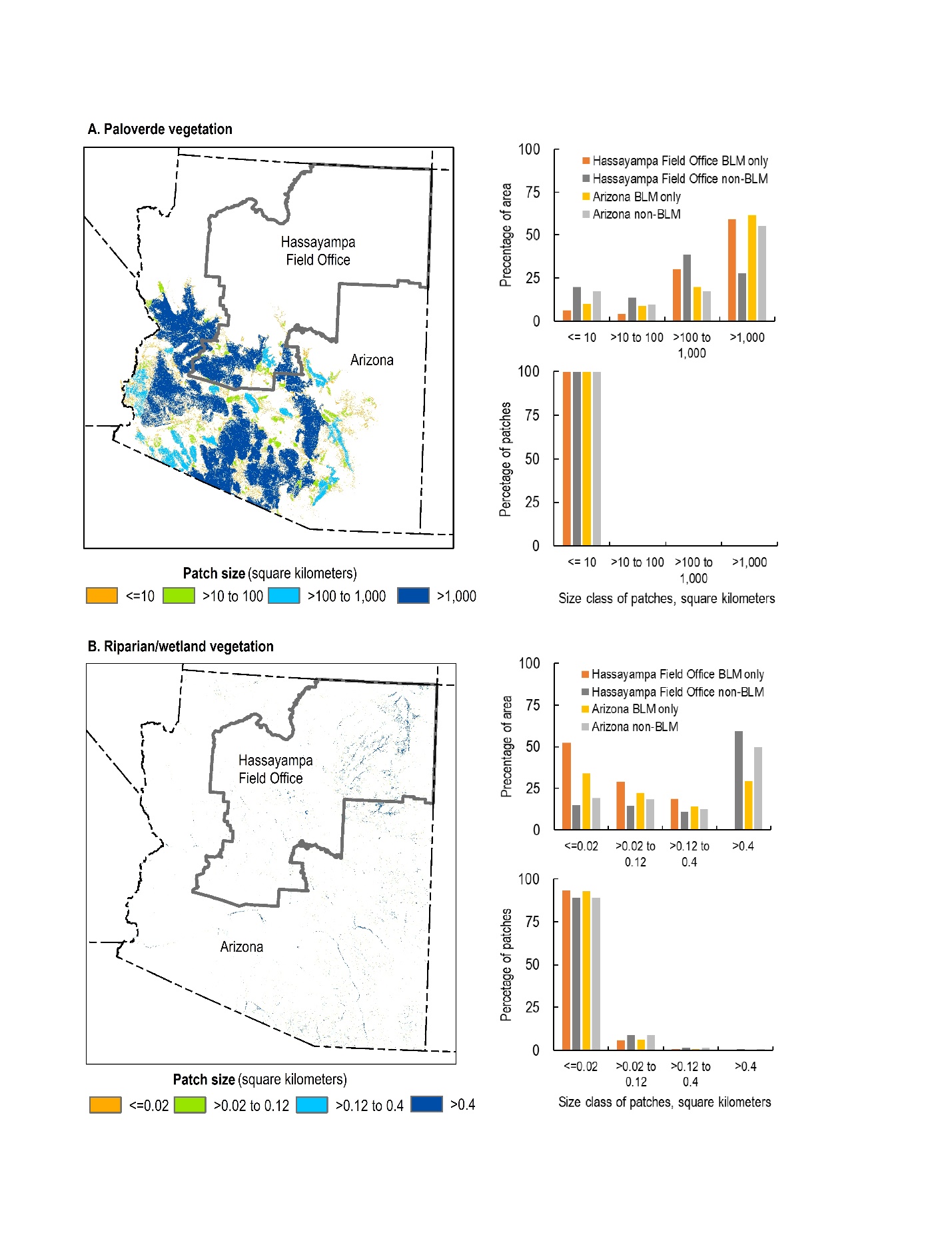


Figure A3. Patch proximity of paloverde mixed cactus vegetation (A, top) and riparian/wetland vegetation (B, bottom) on Bureau of Land Management (BLM)-managed lands and non-BLM lands in the Hassayampa Field Office and Arizona.


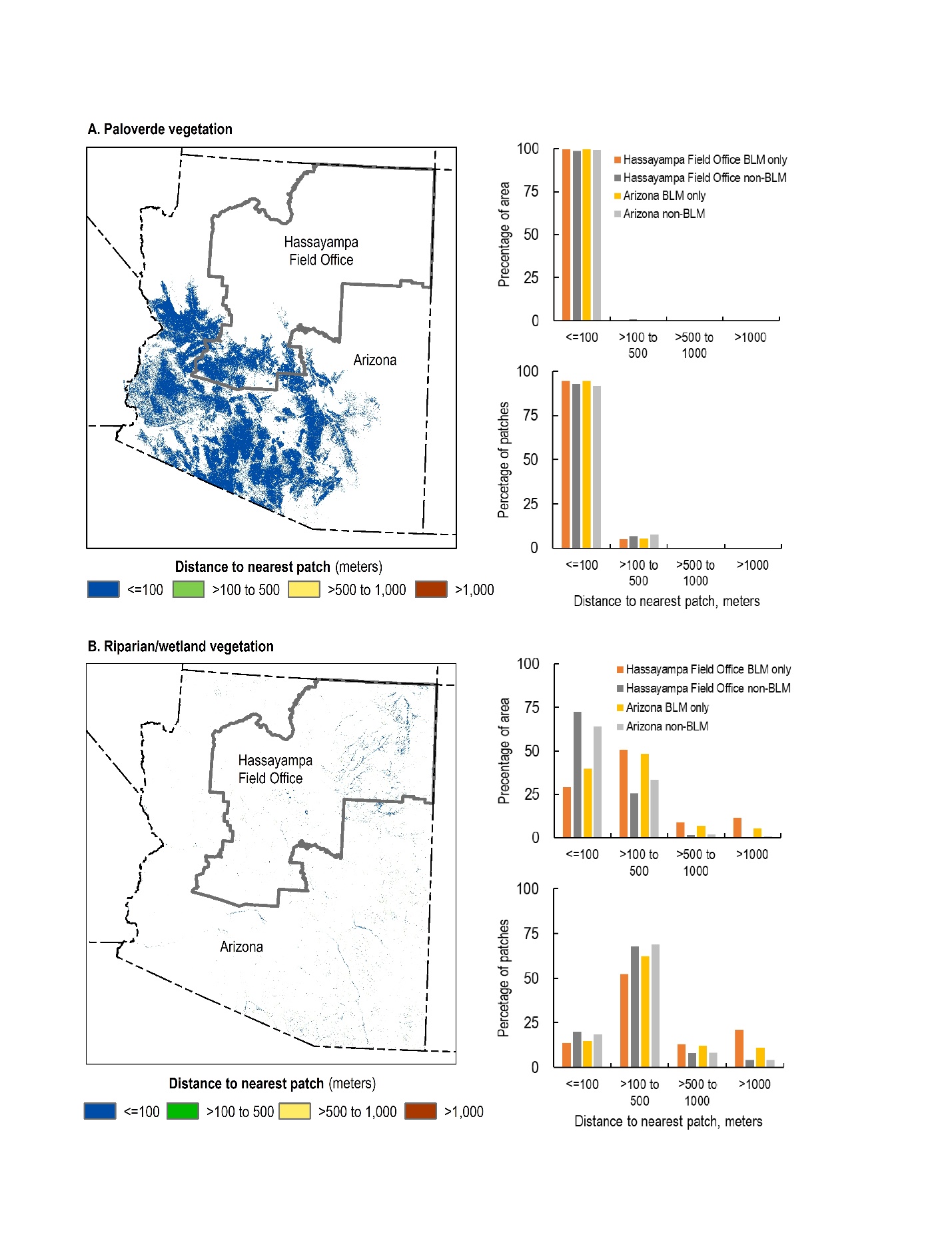


Figure A4. Diversity of current natural vegetation types (A) and change in diversity of natural vegetation types between estimated historic (pre-European settlement) and current vegetation (B) in the Hassayampa Field Office and Arizona. Darker and lighter shades of each color represent Bureau of Land Management (BLM)-managed lands and non-BLM lands, respectively.


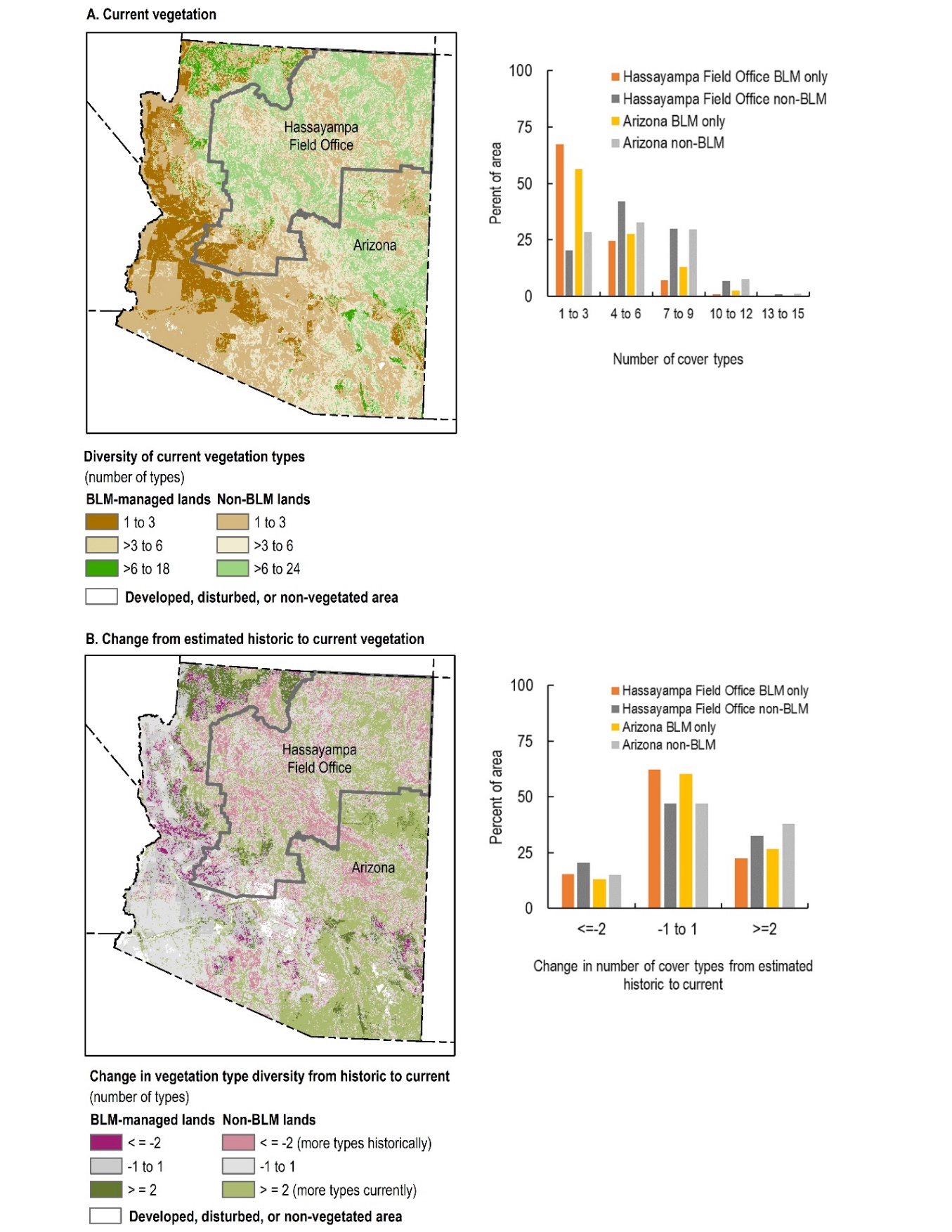


**Appendix B: Eagle Lake Field Office, California**

Figure B1. Priority vegetation types in the Eagle Lake Field Office and State of California. Darker and lighter shades of each color represent presence of the vegetation type on Bureau of Land Management (BLM)-managed lands and on non-BLM lands, respectively.


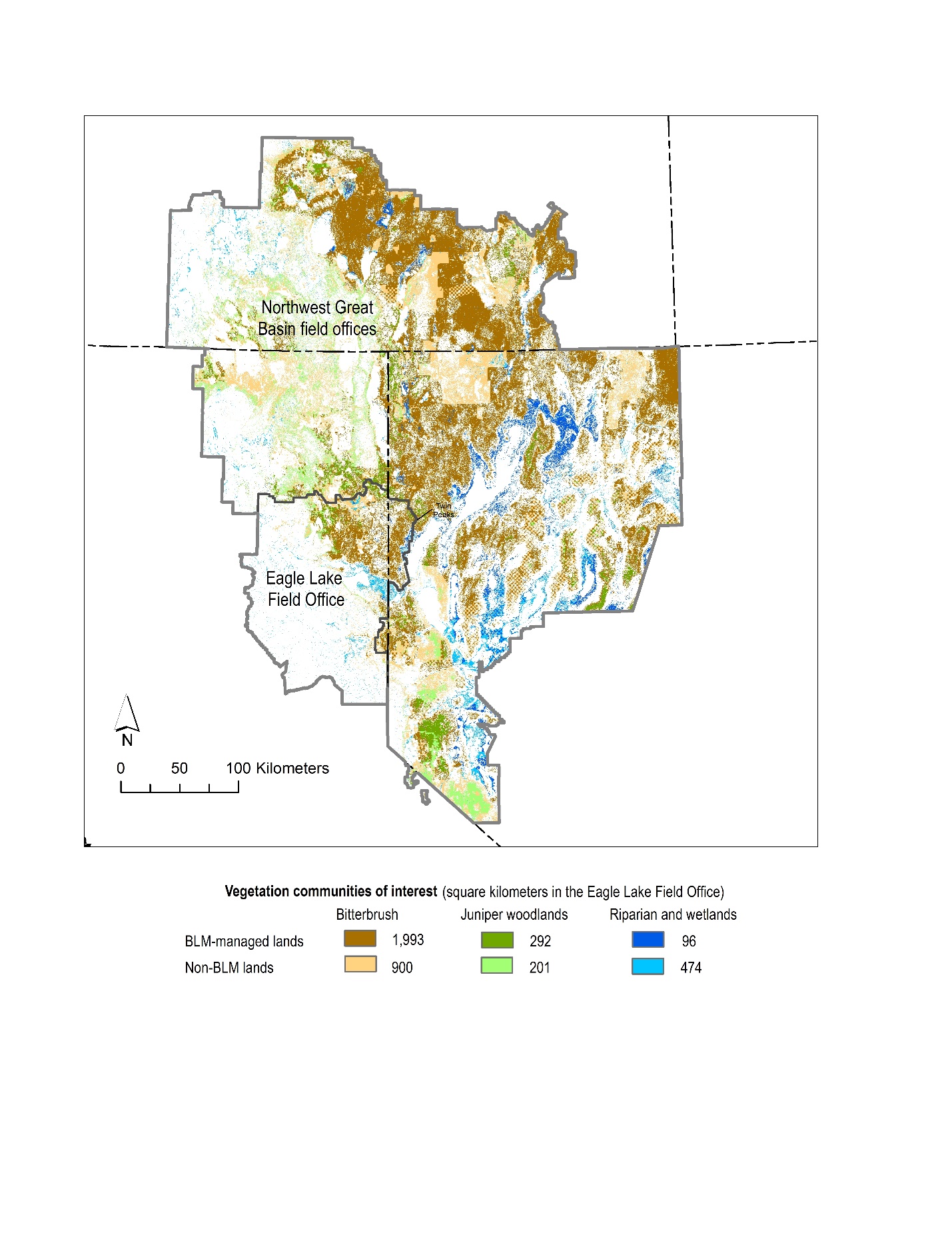


Figure B2. Patch sizes of bitterbrush vegetation (A, top) and riparian/wetland vegetation (B, bottom) on Bureau of Land Management (BLM)-managed lands and on non-BLM lands in the Eagle Lake Field Office and Northwest Great Basin.


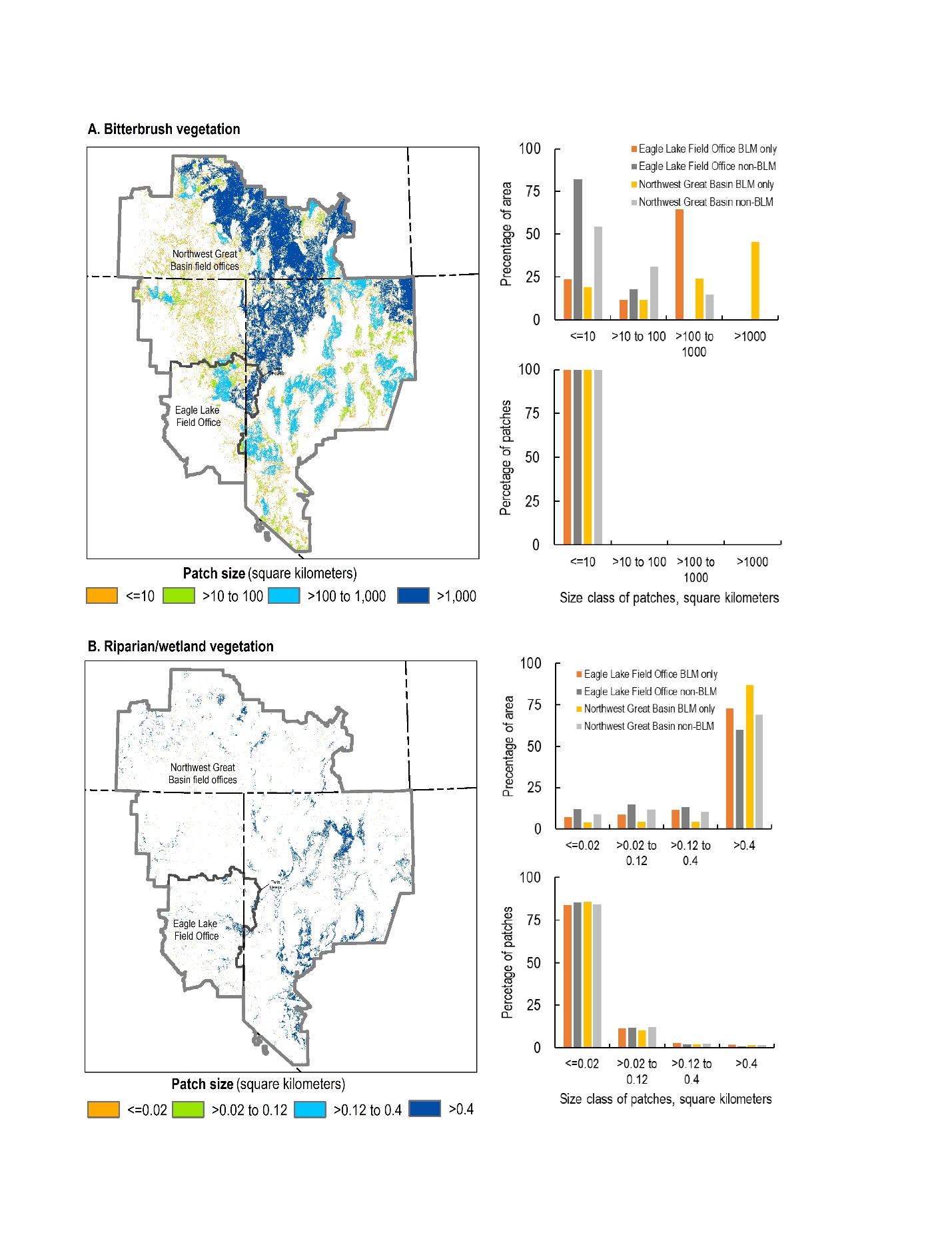


Figure B3. Patch proximity of bitterbrush vegetation (A, top) and riparian/wetland vegetation (B, bottom) on Bureau of Land Management (BLM)-managed lands and non-BLM lands in the Eagle Lake Field Office and Northwest Great Basin.


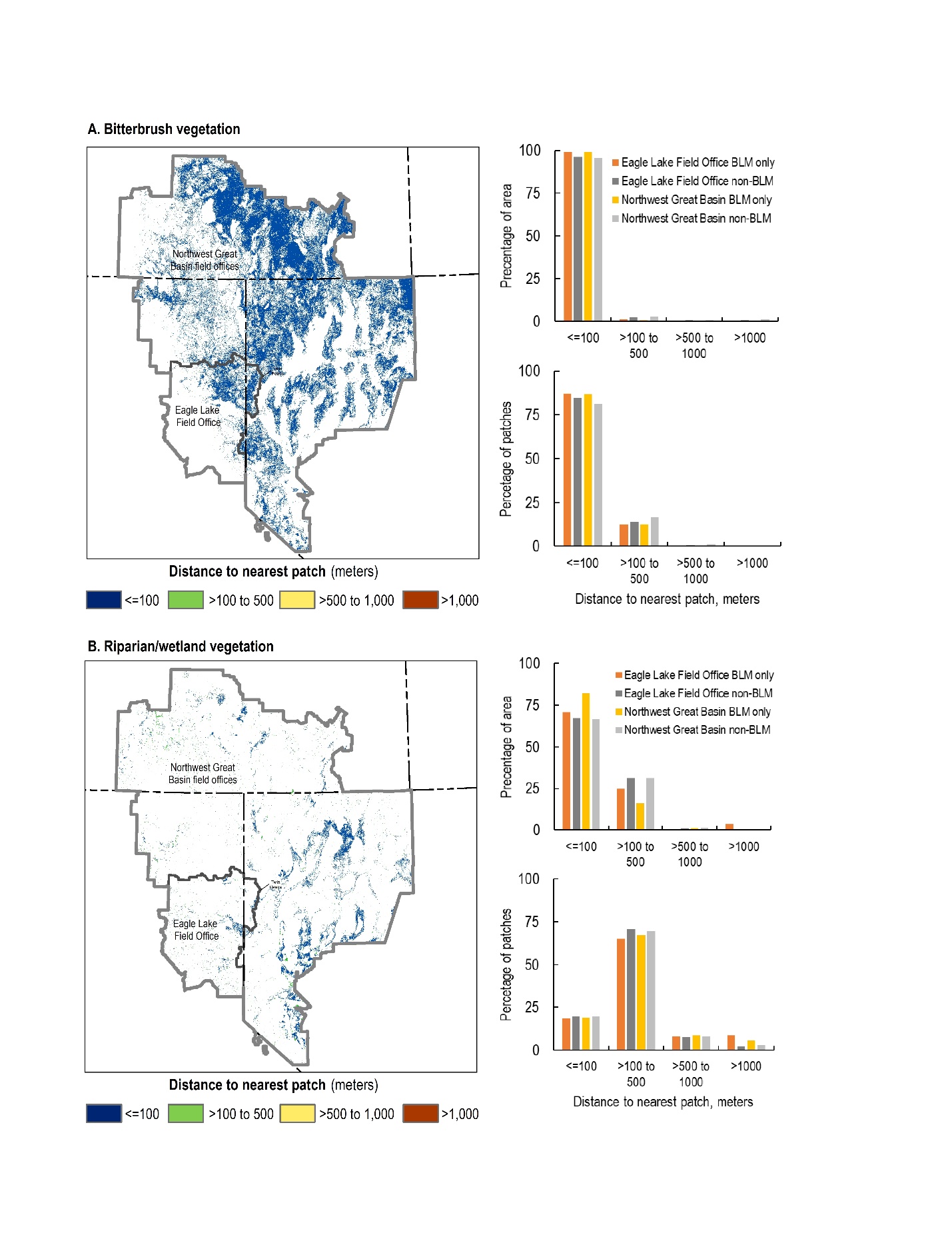


Figure B4. Diversity of current natural vegetation types (A) and change in diversity of natural vegetation types between estimated historic (pre-European settlement) and current vegetation (B) in the Eagle Lake Field Office and Northwest Great Basin. Darker and lighter shades of each color represent Bureau of Land Management (BLM)-managed lands and non-BLM lands, respectively.


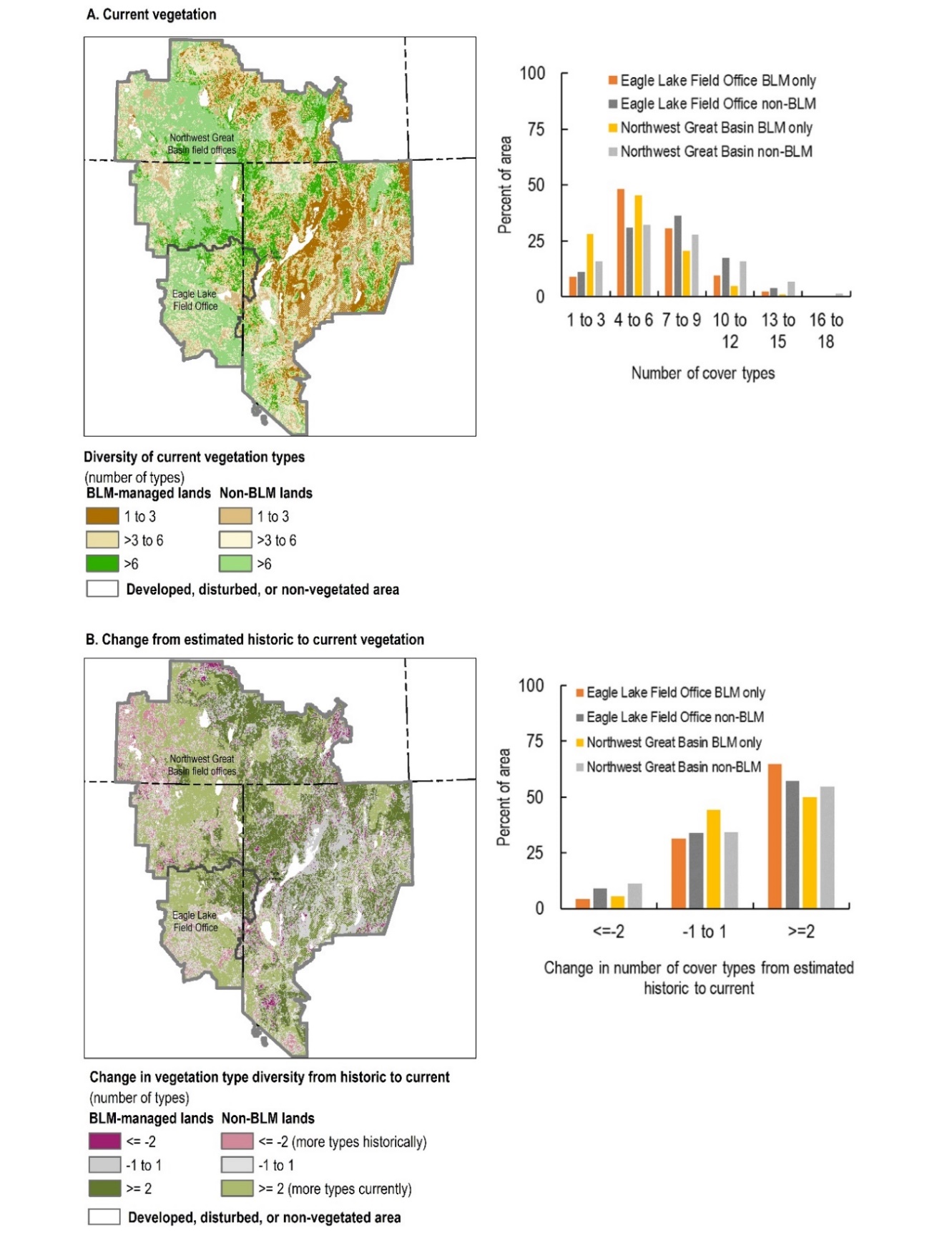


**Appendix C: White River Field Office, Colorado**

Figure C1. Priority vegetation types in a Bureau of Land Management (BLM) grazing allotment and the White River Field Office in Colorado. Darker and lighter shades of each color represent presence of the vegetation type on BLM-managed lands and on non-BLM lands, respectively.


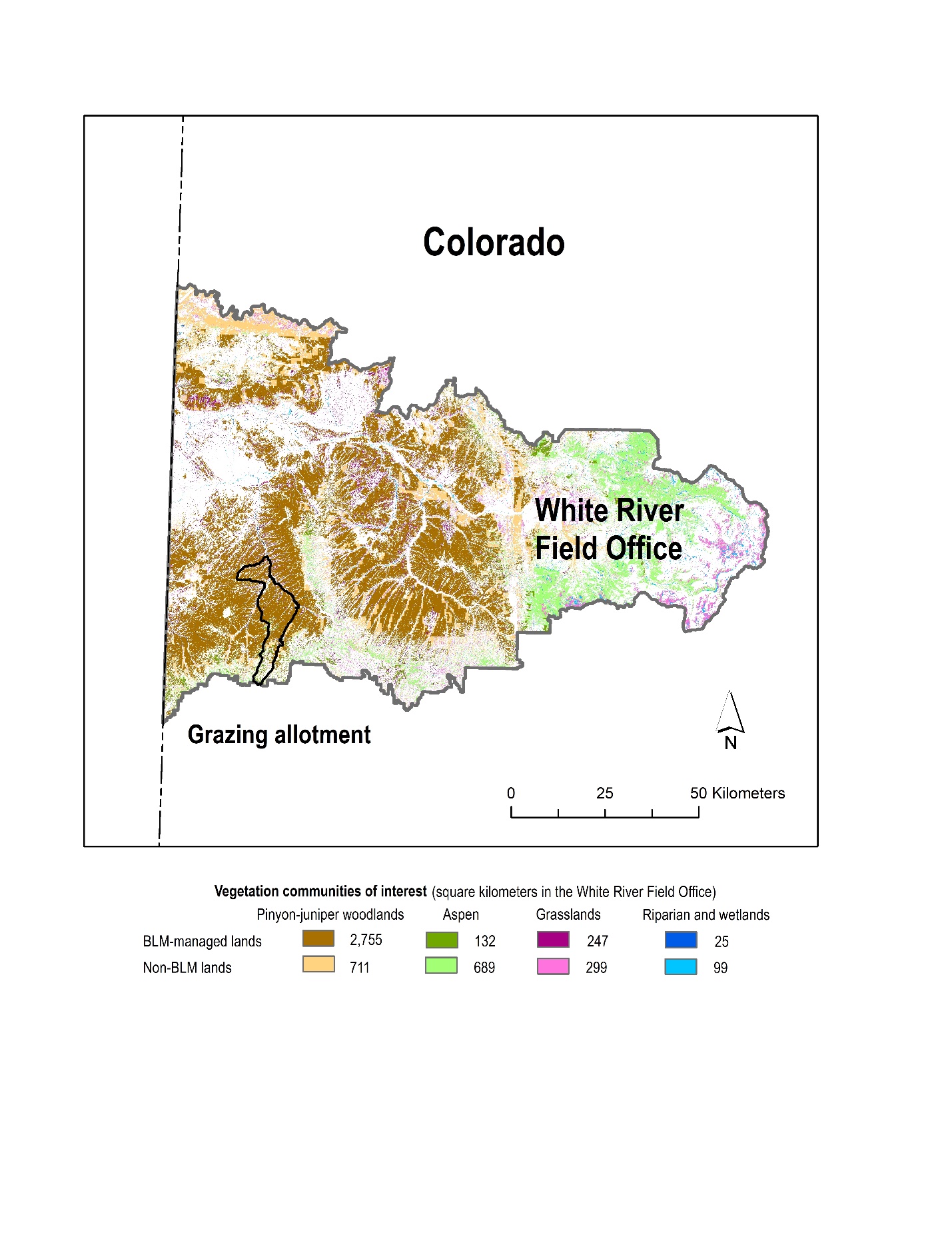


Figure C2. Patch sizes of pinyon-juniper vegetation (A, top) and riparian/wetland vegetation (B, bottom) on Bureau of Land Management (BLM)-managed lands and on non-BLM lands in a BLM grazing allotment and the White River Field Office in Colorado.


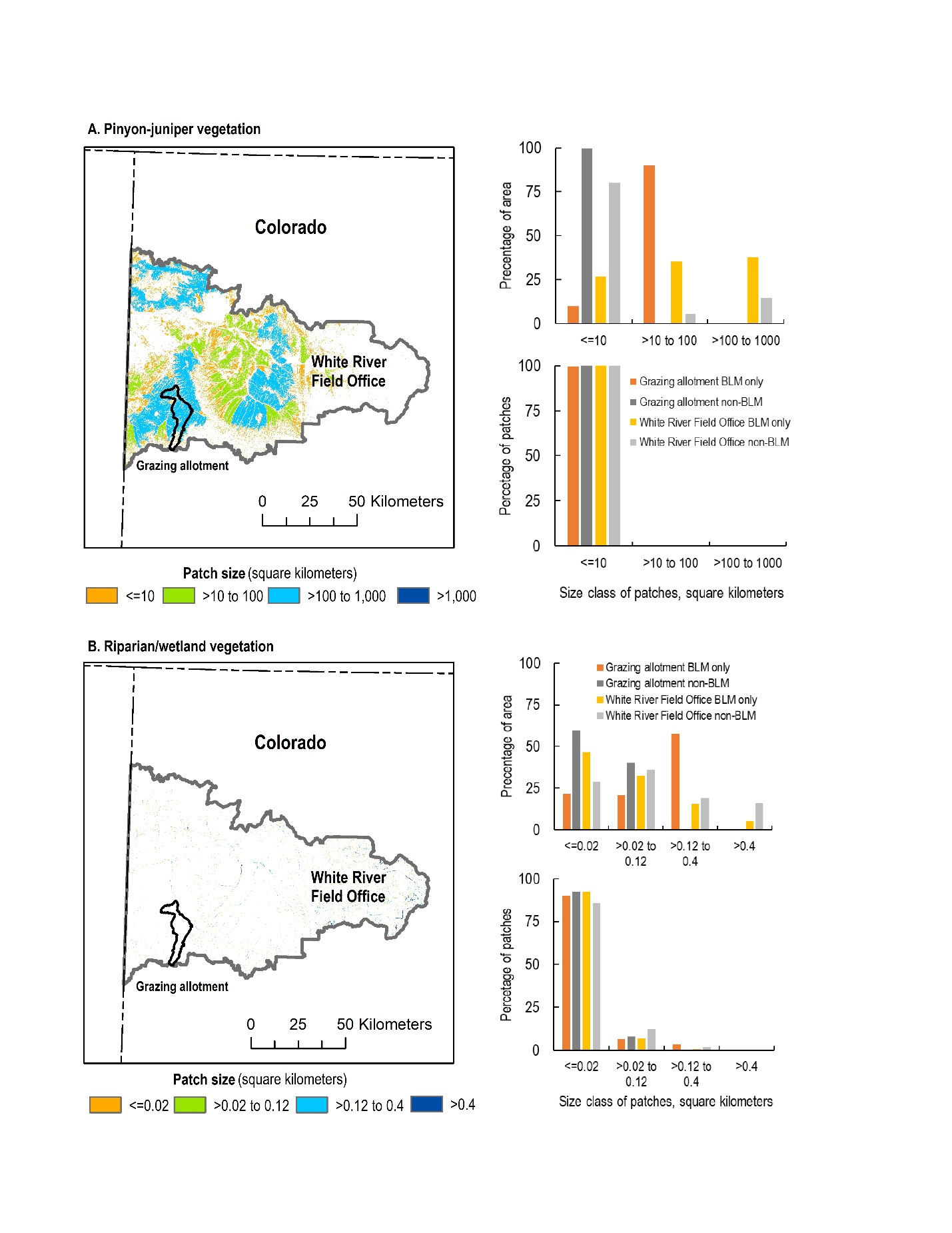


Figure C3. Patch proximity of pinyon-juniper vegetation (A, top) and riparian/wetland vegetation (B, bottom) on Bureau of Land Management (BLM)-managed lands and on non-BLM lands in a BLM grazing allotment and the White River Field Office in Colorado.


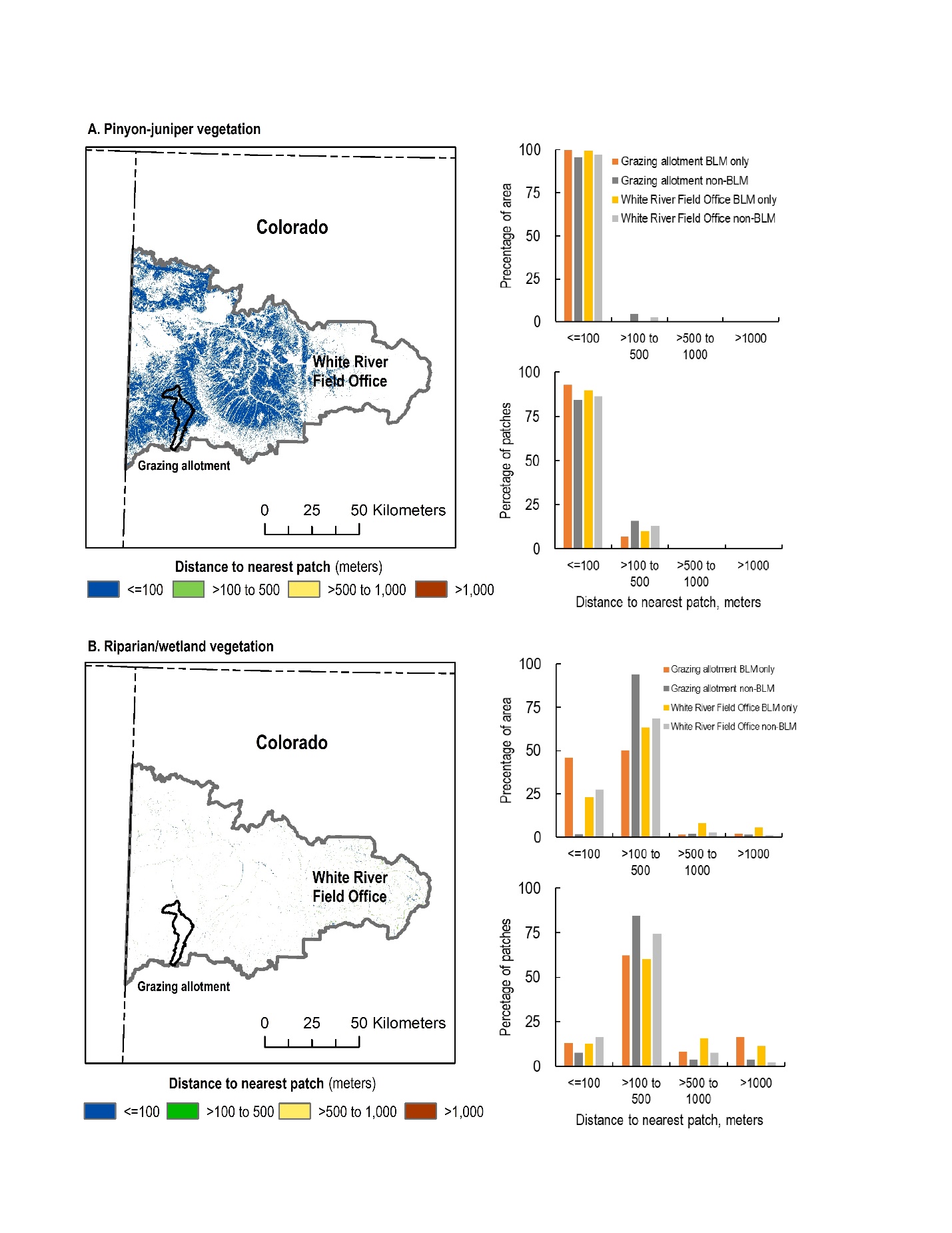


Figure C4. Diversity of current natural vegetation types (A) and change in diversity of natural vegetation types between estimated historic (pre-European settlement) and current vegetation (B) in a Bureau of Land Management (BLM) grazing allotment and the White River Field Office in Colorado. Darker and lighter shades of each color represent BLM-managed lands and non-BLM lands, respectively.


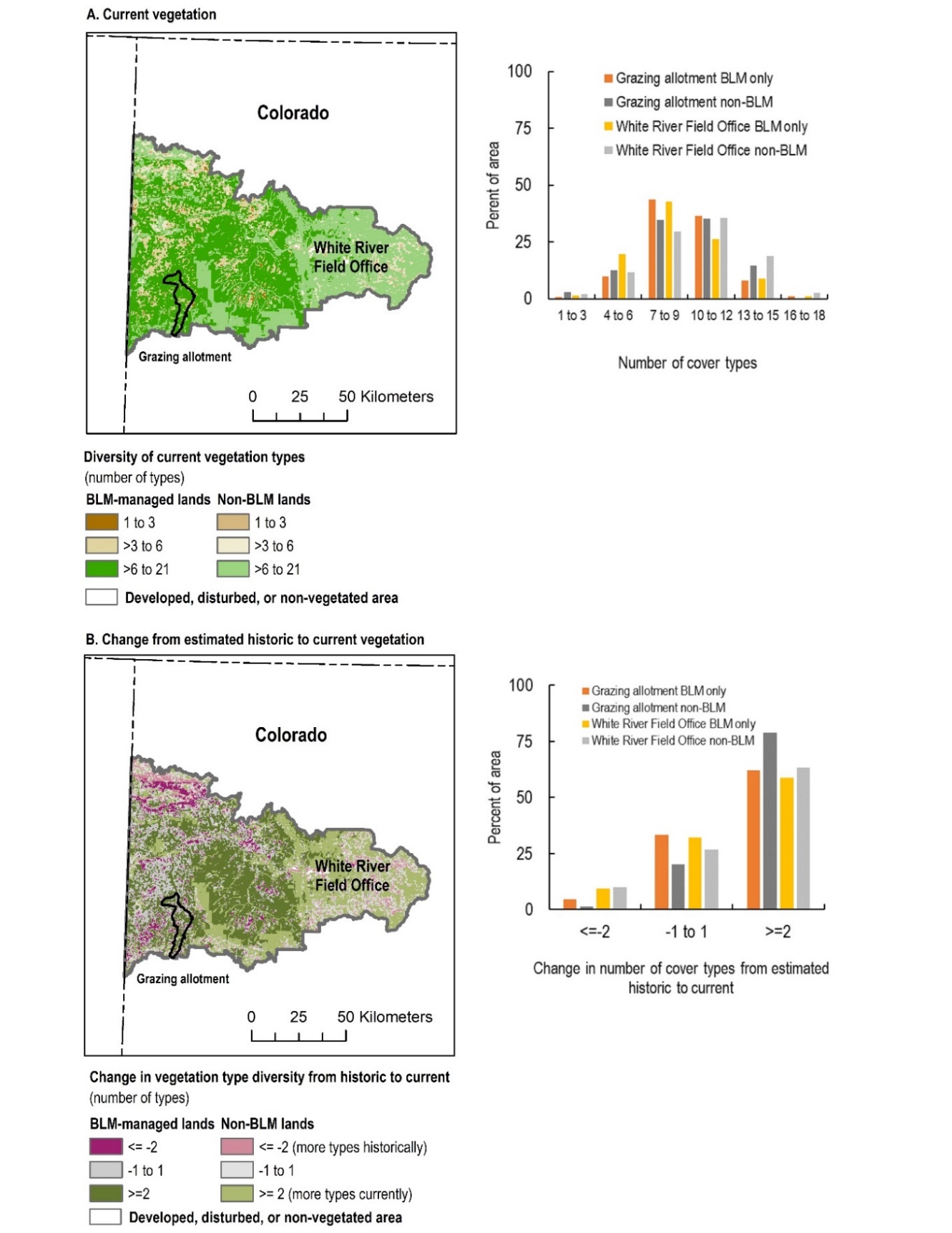

Supplement: Supplementary file 1 — Supplementary Information [file 267_2021_1493_MOESM1_ESM.docx]
